# Systematic Multiomics Analysis of Alterations in *C1QBP* mRNA Expression and Relevance for Clinical Outcomes in Cancers

**DOI:** 10.3390/jcm8040513

**Published:** 2019-04-15

**Authors:** Subbroto Kumar Saha, Kyung Eun Kim, S.M. Riazul Islam, Ssang-Goo Cho, Minchan Gil

**Affiliations:** 1 Department of Stem Cell and Regenerative Biotechnology, Konkuk University, Seoul 05029, Korea; subbroto@konkuk.ac.kr; 2 Department of Cosmetic Sciences, Sookmyung Women’s University, Seoul 04310, Korea; kyungeun@sookmyung.ac.kr; 3 Department of Computer Science and Engineering, Sejong University, Seoul 05006, Korea; riaz@sejong.ac.kr

**Keywords:** *C1QBP*, cancer, patient survival, clinical outcomes, cancer progression, multiomics analysis

## Abstract

*C1QBP* (Complement Component 1 Q Subcomponent-Binding Protein), a multicompartmental protein, participates in various cellular processes, including mRNA splicing, ribosome biogenesis, protein synthesis in mitochondria, apoptosis, transcriptional regulation, and infection processes of viruses. The correlation of *C1QBP* expression with patient survival and molecular function of *C1QBP* in relation to cancer progression has not been comprehensively studied. Therefore, we sought to systematically investigate the expression of *C1QBP* to evaluate the change of *C1QBP* expression and the relationship with patient survival and affected pathways in breast, lung, colon, and bladder cancers as well as lymphoma. Relative expression levels of *C1QBP* were analyzed using the Oncomine, Gene Expression Across Normal and Tumor Tissue (GENT), and The Cancer Genome Atlas (TCGA) databases. Mutations and copy number alterations in *C1QBP* were also analyzed using cBioPortal, and subsequently, the relationship between *C1QBP* expression and survival probability of cancer patients was explored using the PrognoScan database and the R2: Kaplan Meier Scanner. Additionally, the relative expression of *C1QBP* in other cancers, and correlation of *C1QBP* expression with patient survival were investigated. Gene ontology and pathway analysis of commonly differentially coexpressed genes with *C1QBP* in breast, lung, colon, and bladder cancers as well as lymphoma revealed the *C1QBP*-correlated pathways in these cancers. This data-driven study demonstrates the correlation of *C1QBP* expression with patient survival and identifies possible *C1QBP*-involved pathways, which may serve as targets of a novel therapeutic modality for various human cancers.

## 1. Introduction

Cancer is one of the leading causes of death, and also an increasing threat to human health worldwide [1,2]. A total of 17.5 million new cancer cases and 8.7 million cancer deaths were estimated in 2015 [2]. In addition, the number of incident cases of cancer is increasing due to population growth, an aging population, and increasing age-specific incident rates [2]. Many efforts in cancer prevention, early diagnosis, and curation have been invested to reduce the cancer burden. Accumulation of gene alterations is crucial to oncogenesis and closely related to the prognosis of cancer patients. Identification of differentially expressed genes that are associated with survival in cancer patients can be utilized as diagnostic markers for early diagnosis of cancers. Moreover, an understanding of the mechanism of the altered expression of these genes will enable them to be exploited as therapeutic targets. 

*C1QBP* (Complement Component 1 Q Subcomponent-Binding Protein) is a multifunctional acidic protein, distributed among multiple organelles including mitochondria, cell surface, cytosol, and nucleus [3,4,5,6,7]. *C1QBP* is found to have the highest expression in the mitochondria, where it is thought to have a role in protein synthesis [8]. It may be involved in the ribosome maturation process and RNA splicing [9,10]. On the cell surface, *C1QBP* functions as a receptor of multiple ligands including C1q, high molecular weight kininogen, and coagulation factor XII, which are involved in inflammation and innate immunity [3,11,12,13,14,15]. In the nucleus, *C1QBP* forms complexes with some transcription factors and modulates transcriptional activities by interacting with the transcription factors [16,17,18]. *C1QBP* is also involved in the pathogenesis of infectious microbes by binding to the carbohydrates or proteins of bacteria or viruses [19,20,21,22,23]. 

*C1QBP* may play oncogenic roles in various cancers. Higher expression of *C1QBP* has been related to poorer clinical outcomes in breast [24,25], ovarian [26], endometrial [27], and cervical [28] cancers. Expression of *C1QBP* is also associated with proliferation and metastasis in breast cancer cells [29,30]. Moreover, ectopic expression of *C1QBP* enhances metastasis in melanoma cells [31]. Knockdown of *C1QBP* reduces lamellipodia formation and cancer metastasis in lung carcinoma cells [32]. In contrast, overexpression of *C1QBP* represses metastasis of renal carcinoma cells [33]. These findings suggest that *C1QBP* plays important roles in the progression of multiple cancers. 

To investigate the expression of *C1QBP* and evaluate the possible prognostic value of *C1QBP* for targeting various cancers, we systematically analyzed the *C1QBP* expression and its clinical outcomes in certain cancers with numerous expression and patient survival datasets, available in various recognized online platforms. We also investigated the genes usually co-altered with *C1QBP* with respect to the five cancer types with high *C1QBP* expression. Thus, these analyses might reveal the value of *C1QBP* expression for patient survival and provide a realization of the possible underlying mechanism of human cancers, which might bear a potential implication in *C1QBP*-targeted cancer therapy. 

## 2. Experimental Section

### 2.1. Oncomine Database Analysis

The mRNA expression level of *C1QBP* in various cancer tissues and their normal cell counterparts was obtained from the Oncomine database version 4.5 (Thermo Fisher Scientific Inc., Ann Arbor, MI, USA) (https://www.oncomine.org/resource/login.html) [34,35]. The fold-change of mRNA expression in cancers compared to that in their normal counterparts was calculated. The statistical significance of differences was determined by *p*-value, generated by Student’s *t*-test with threshold <1E−4. The results are listed in Tables S1, S3, S5, and S7.

### 2.2. Analysis Using the Gene Expression Across Normal and Tumor Tissue (GENT) Database and UALCAN 

*C1QBP* mRNA expression in various types of cancer and normal counterparts was examined in the GENT database (Korea Research Institute of Bioscience and Biotechnology, Daejeon, Korea) (http://medical-genome.kribb.re.kr/GENT/) [36], the UALCAN (Preston, Lancashire, UK) (http://ualcan.path.uab.edu/index.html) [37], the gene expression profiling interactive analysis (GEPIA) (Beijing, China) (http://gepia.cancer-pku.cn/) [38] and TCGA Wanderer (Badalona, Spain) (http://maplab.imppc.org/wanderer/) [39]. The query with *C1QBP* was carry out in default setting to obtain their respective expression pattern derived from the datasets profiled by Affymetrix U133A (GENT database) and The Cancer Genome Atlas (TCGA) datasets (ULCAN, GEPIA, and TCGA wanderer).

### 2.3. cBioPortal Database Analysis

We carried out the altered expression analysis of *C1QBP* in various cancers using the cBioPortal database version 2.2.0 (Center for Molecular Oncology at MSK, New York, NY, USA) (http://www.cbioportal.org/) [40,41], an open access web-based resource, currently providing data from 225 cancer studies in TCGA pipeline. Altered frequencies of mRNA expression were estimated by subtypes of each cancer from TCGA PanCanAtlas dataset. Somatic copy number alterations within the portal are generated by the GISTIC (Genomic Identification of Significant Targets in Cancer) algorithm. Expression of *C1QBP* was examined by each alteration status (deep deletion, shallow deletion, diploid, gain, and amplification) and plotted. Statistical analysis was performed by ANOVA and unpaired *t*-test using GraphPad 7 software (GraphPad Software, Inc., San Diego, CA, USA).

### 2.4. SurvExpress Biomarker Validation Tool

Expression of *C1QBP* in high and low risk groups, derived from 502 patient samples in TCGA dataset, was evaluated by SurvExpress biomarker validation tool version 2.0 (Monterrey, Nuevo Leon, Mexico) (http://bioinformatica.mty.itesm.mx:8080/Biomatec/SurvivaX.jsp) [42]. The prognostic index of each sample was estimated by Cox survival analysis. A total of 251 patient samples that had a higher prognostic index than the other 251 samples were classified as the high-risk group; the other half of the samples was classified as the low-risk group. *C1QBP* expression of each risk group was graphed into box plots.

### 2.5. PrognoScan Database Analysis

The relation between the expression of *C1QBP* and patient prognosis in various types of cancers was investigated using the PrognoScan database (Chūō, Tokyo, Japan) (http://dna00.bio.kyutech.ac.jp/PrognoScan/). The statistical significance was determined with threshold of a Cox *p*-value < 0.05. The results are summarized in Tables S2, S4, S6, and S8.

### 2.6. R2: Kaplan Meier Scanner

The R2: Genomics Analysis and Visualization Platform version 3.2.0 (http://r2.amc.nl) is a web-based genomics analysis tool developed by Jan Koster at the Academic Medical Center, Amsterdam (the Netherlands). We carried out survival analysis of cancer patients with the R2: Kaplan Meier Scanner by *C1QBP* gene expression. A cutoff between high expression and low expression groups was selected, where *p*-values obtained from the log-rank test were minimized.

### 2.7. Finding Coexpressed Genes of C1QBP and Its Pathway Analysis

Positively and negatively coexpressed genes of *C1QBP* were explored in TCGA dataset of five different cancers (breast, colon, lung, bladder cancers, and lymphoma), using the R2: Genomics Analysis and Visualization Platform version 3.2.0 (Academic Medical Center, Amsterdam, Netherlands) (https://hgserver1.amc.nl/), with the adjustment of Bonferroni test and cutoff *p*-value was selected as <0.01. Thereafter, the common gene set was explored by drawing Venn diagrams, using coexpressed genes from five different cancers. 

To explore pathways and gene ontology shared by *C1QBP*-correlated genes, we used Reactome analysis tool version 67.0 (Hinxton, Cambridge, UK) (https://reactome.org/) [43] and GOTermFinder functional annotation tool version 1.0 (Princeton, NJ, USA) (https://go.princeton.edu/cgi-bin/GOTermFinder) [44] and subsequently classified them based on their KEGG (Kyoto Encyclopedia of Genes and Genomes) pathway. 

## 3. Results

### 3.1. C1QBP Expression in Various Cancers

**Figure 1:**
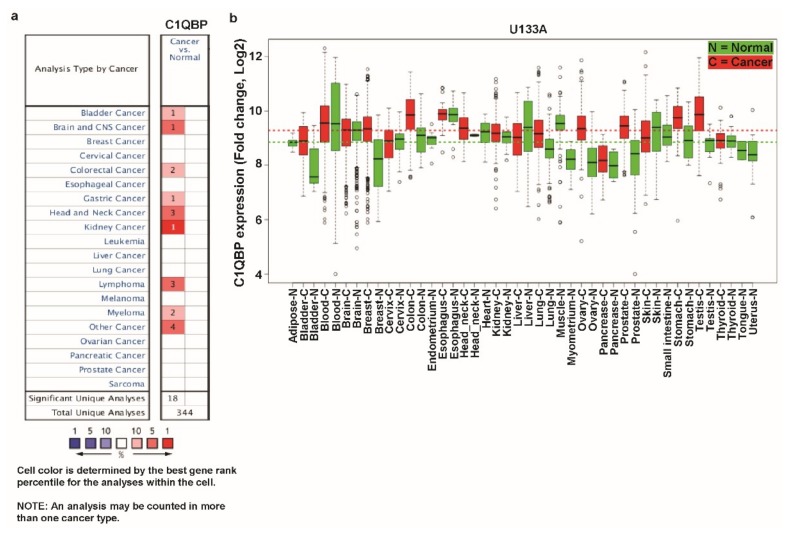
Transcription levels of *C1QBP* (Complement Component 1 Q Subcomponent-Binding Protein) in different types of cancer using the Oncomine and Gene Expression Across Normal and Tumor Tissue (GENT) databases. (**a**) This graphic was generated from the Oncomine database (available at https://www.oncomine.org/resource/login.html), indicating the number of datasets with statistically significant (*p* < 0.01) mRNA over-expression (red) or under-expression (blue) of *C1QBP* (different types of cancer vs. corresponding normal tissue). The threshold was designed with following parameters: *p*-value of 1e−4, fold change of 2, and gene ranking of 10%. (**b**) Expression pattern of *C1QBP* mRNA in normal and tumor tissues. *C1QBP* mRNA expression in various types of cancer was searched in the GENT database (available at http://medical-genomics.kribb.re.kr/GENT/). Boxes represent the median and the 25th and 75th percentiles; dots represent outliers. Red boxes represent tumor tissues; green boxes represent normal tissues. Red and green dashed lines represent the average value of all tumor and normal tissues, respectively. Abbreviations. CNS: central nervous system.

To examine the differential expression level of *C1QBP* in various cancers and their counterparts, we utilized the Oncomine and GENT databases. In the Oncomine database, we queried with “*C1QBP*” using the default threshold parameters: *p*-value of 1e−4, fold change of 2, and gene ranking of 10%. Compared to the expression level in the normal counterpart, expression of *C1QBP* was upregulated in bladder, brain and central nervous system (CNS), colorectal, gastric, head and neck and kidney cancers, as well as lymphoma, myeloma, and some other cancers (Figure 1a). There was no downregulated expression of *C1QBP* in all types of cancers in this analysis. GENT provides the respective expression data over various kinds of cancers and their normal counterparts based on the microarray data profiled by Affymetrix platforms. In databases using U133 platform, *C1QBP* expression is upregulated in certain cancer types including bladder, breast, colon, lung, prostate, stomach, and testis cancers (Figure 1b). The average expression of *C1QBP* was higher in cancer tissues than in the normal tissues in the analysis using the GENT database. Analysis with the Oncomine and GENT databases showed that expression of *C1QBP* was apparently augmented in multiple cancers including the most common breast, lung, and colon cancers. Therefore, we chose breast, lung, and colon cancers; in addition, bladder cancers and lymphoma were chosen among the other cancers in which *C1QBP* expression was higher than in normal tissue for further systematic expression and prognosis analysis. 

### 3.2. C1QBP Expression Pattern and Patient Survival in Breast Cancer.

**Figure 2:**
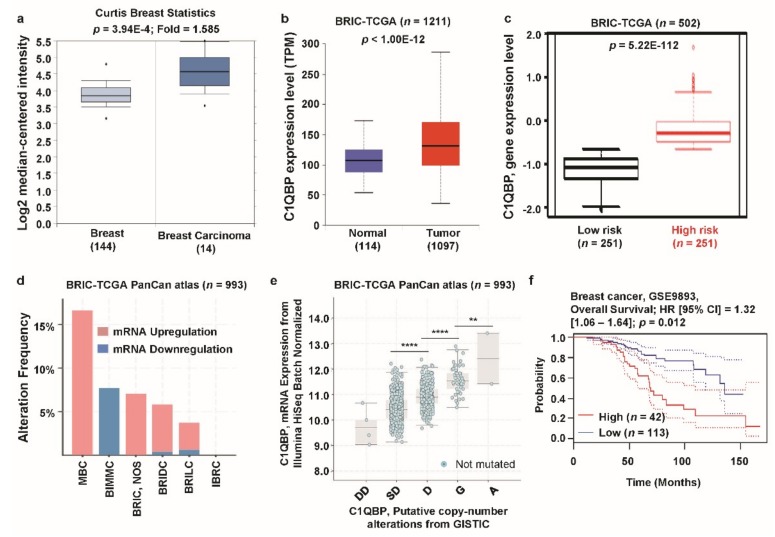
*C1QBP* expression pattern and patient survival analysis in breast cancer compared to *C1QBP* expression in normal tissue and cancer tissue. (**a**) The fold-change of *C1QBP* in breast cancers was identified by our analyses, shown as a box plot. The box plot comparing specific *C1QBP* expression in normal (*n* = 144, left plot) and cancer tissue (*n* = 14, right plot) was derived from the Oncomine database. The analysis was shown in breast carcinoma relative to in normal breast. The asterisk above and below the box represent maximum and minimum value, respectively. (**b**) Expression of *C1QBP* gene in The Cancer Genome Atlas (TCGA) database. Box plots showing the *C1QBP* mRNA expression in BRIC tumor (red plot) and their normal (blue plot) tissues, using data from the TCGA database through ULCAN. (**c**) *C1QBP* gene expression in BRIC patients from TCGA database. The Box-plots generated using SurvExpress biomarker validation tool showing the gene expression in BRIC patients using cohorts from datasets generated by TCGA (*n* = 502). Box-plots show expression levels and *p*-values resulting from the *t*-test of the difference expression between high risk (red) and low risk (green) groups in BRIC patients. (**d**) Alterations (mRNA upregulation and downregulation) of the *C1QBP* gene in BRIC (TCGA, PanCan atlas) (*n* = 993). Data were obtained using cBioPortal. (**e**) *C1QBP* mRNA expression was significantly associated with the copy number alteration status, deep deletion (DD), shallow deletion (SD), diploid (D), gain (G), and amplification (A) (ANOVA, *p* < 0.0001) (**: *p* < 0.01; ****: *p* < 0.0001). (**f**) The survival curve comparing patients with high (red) and low (blue) expression in breast cancer was plotted from the PrognoScan database. Survival curve analysis was conducted using a threshold Cox *p*-value < 0.05. Abbreviations. MBC: metaplastic breast cancer; BIMMC: breast invasive mixed mucinous carcinoma; BRIC: breast invasive carcinoma; BRIDC: breast invasive ductal carcinoma; BRILC: breast invasive lobular carcinoma; IBRC: invasive breast carcinoma.

To examine the expression of *C1QBP* in breast cancer and their corresponding normal counterparts, we analyzed datasets in the Oncomine and TCGA database. The relative expression of *C1QBP* in 144 primary breast tissues versus 14 breast carcinomas was analyzed in the Curtis dataset. *C1QBP* expression was significantly upregulated in breast carcinomas, compared to the normal breast tissue (*p* = 3.94E−4, Figure 2a). In the Curtis dataset, all types of breast cancers were found to have significantly higher expression of *C1QBP* than their normal counterparts (Supplementary Table S1). Expression of *C1QBP* gene was also significantly (*p* < 1.00E−12) higher in breast invasive carcinoma (BRIC) than the normal counterpart in TCGA database (Figure 2b). To analyze the association of *C1QBP* expression with patients’ risk, we determined the expression of *C1QBP* in high- and low-risk groups, derived from 502 patient samples in the TCGA dataset by the SurvExpress biomarker validation tool [40]. The expression of *C1QBP* mRNA was significantly augmented in the high-risk group (*p* = 5.22E−112) (Figure 1c). Next, alterations in *C1QBP* mRNA expression were found in the BRIC TCGA PanCanAtlas dataset (Figure 2d). Among breast cancer types, metaplastic breast cancer (MBC) had the highest alteration frequency (17.0%). Upregulation of *C1QBP* was found to be the most frequent alteration type in metaplastic breast cancer (MBC), breast invasive carcinoma (BRIC), breast invasive ductal carcinoma (BRIDC), and breast invasive lobular carcinoma (BRILC). However, breast invasive mixed mucinous carcinoma (BIMMC) only showed down-regulation of *C1QBP* mRNA expression. To determine whether *C1QBP* copy number status correlated with mRNA expression, we determined *C1QBP* mRNA expression in each case showing copy number alteration. *C1QBP* expression was positively associated with the copy number alteration status, significantly (ANOVA, *p* < 0.0001) (Figure 2e). In addition, we compared patient survival with *C1QBP* expression from the PrognoScan database (Supplementary Table S2). In the GSE9893 dataset, patients with high expression of *C1QBP* (*n* = 42) had significantly lower overall survival compared to patients with lower expression of *C1QBP* (*n* = 113) (Figure 1f). Overall, these data-driven results suggest that expression of *C1QBP* is significantly upregulated in breast cancer cells and is positively correlated with patient poor survival.

### 3.3. C1QBP Expression Pattern and Patient Survival in Lung Cancer

**Figure 3:**
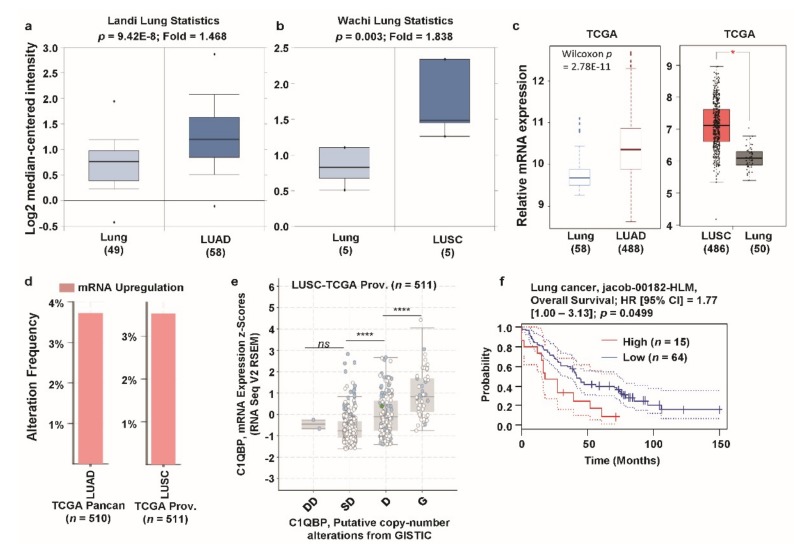
*C1QBP* expression pattern and patient survival analysis in lung cancer: comparison between *C1QBP* expression in normal tissue and cancer tissue. (**a**) The fold-change of *C1QBP* in lung cancer was identified by our analyses, shown as a box plot. The box plot comparing specific *C1QBP* expression in normal (*n* = 49, left plot) and cancer tissue (*n* = 58, right plot) was derived from the Oncomine database. The analysis compared expression in LUAD, relative to expression in normal lung. The asterisk above and below the box represent maximum and minimum value, respectively. (**b**) The fold-change of *C1QBP* in lung cancers was identified by our analyses, shown as a box plot. The box plot comparing specific *C1QBP* expression in normal (*n* = 5, left plot) and cancer tissue (*n* = 5, right plot) was derived from the Oncomine database. The analysis shown is of the expression in LUSC relative to that in normal lung. (**c**) Expression of *C1QBP* gene in The Cancer Genome Atlas (TCGA) database. Box plots showing the *C1QBP* mRNA expression in LUSC tumor (T, red plot) and the corresponding normal (N, gray plot) tissues, using data from the TCGA database through TCGA Wanderer and GEPIA. *: *p* < 0.01. (**d**) Alterations (mRNA upregulation) of the *C1QBP* gene in LUAD (TCGA PanCanAtlas; *n* = 510) and LUSC (TCGA Provisional; *n* = 511). Data was obtained using cBioPortal. (**e**) *C1QBP* mRNA expression was significantly associated with the copy number alteration status (ANOVA, *p* <0.0001) in lung cancer. (****: *p* < 0.0001; *ns*: nonsignificant) (**f**) The survival curve comparing patients with high (red) and low (blue) expression in lung cancer was plotted from the PrognoScan database. Survival curve analysis was conducted using a threshold Cox *p*-value <0.05. Abbreviations. LUAD: lung adenocarcinoma; LUSC: lung squamous cell carcinoma.

Next, we focused on lung cancer due to the high expression of *C1QBP* according to the GNET database (see Figure 1b). Previously, high expression of *C1QBP* was also reported in lung cancer cells and tissues [45,46]. However, systematic analysis of the correlation between *C1QBP* expression and patient survival has not been carried out to date. To investigate the expression level of *C1QBP* in clinical specimens, we analyzed the microarray datasets of lung cancer and normal counterparts, using the Oncomine database (Figure 3a,b, Supplementary Table S3). Expression of *C1QBP* was significantly upregulated in all available datasets for lung cancer in the Oncomine database (Supplementary Table S3). Upregulation of *C1QBP* (1.468-fold) was observed in lung adenocarcinoma (LUAD) of the Landi dataset and a 1.838-fold increase was observed in lung squamous cell carcinoma (LUSC) of Wachi dataset (shown in Figure 2a,b, as representative data). Expression of *C1QBP*, analyzed using the TCGA database through TCGA Wanderer and GEPIA, was found to be significantly increased in LUAD and LUSC compared to the normal lung tissues (Figure 3c). The proportion of genetic alterations (predominantly upregulation) in the *C1QBP* gene in LUAD (TCGA PanCanAtlas dataset) and LUSC (TCGA Provisional dataset) was around 4% (Figure 3d). *C1QBP* mRNA expression showed a significant positive correlation with the copy number alteration status in LUSC (analysis based on TCGA Provisional dataset) (Figure 3e). The patient group with a high expression level of *C1QBP* mRNA showed significantly poor overall survival compared to the low expression group, as revealed by the analysis of the jacob-00182-HLM dataset, accessed from thePrognoScan database (Figure 3f, Supplementary Table S4). Therefore, these results suggest that *C1QBP* expression, owing to copy number alterations, is upregulated in lung cancer tissues, and is positively correlated with patient poor survival.

### 3.4. C1QBP Expression Pattern and Patient Survival in Colon Cancer

**Figure 4:**
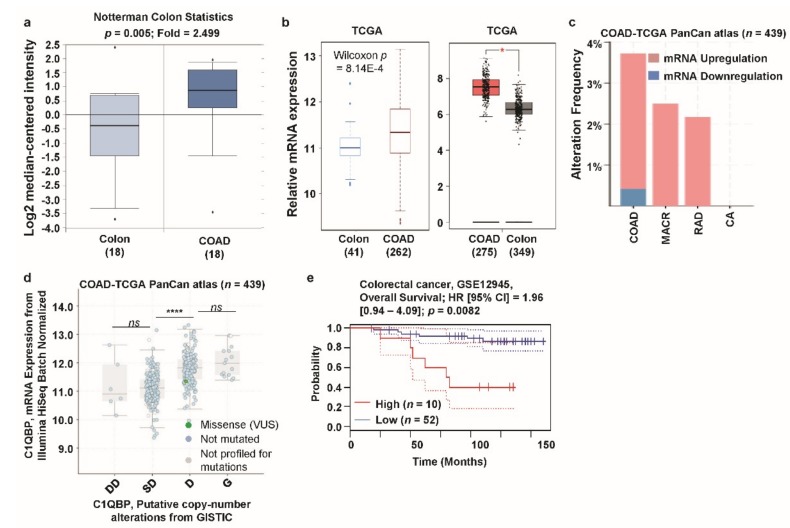
*C1QBP* expression pattern and patient survival analysis in colon cancer: comparison of *C1QBP* expression in normal tissue and cancer tissue. (**a**) The fold-change of *C1QBP* in colon cancers was identified by our analyses, represented as a box plot. The box plot comparing specific *C1QBP* expression in normal (*n* = 18, left plot) and cancer tissue (*n* = 18, right plot) was derived from the Oncomine database. The analysis was performed in COAD in comparison to normal colon. The asterisk above and below the box represent maximum and minimum value, respectively. (**b**) Expression of *C1QBP* gene in the Cancer Genome Atlas (TCGA) database. Box plots showing the *C1QBP* mRNA expression in COAD tumors (T, red plot) and the respective normal (N, gray/blue plot) tissues, using data from the TCGA database through TCGA Wanderer and Gene Expression Profiling Interactive Analysis (GEPIA). *: *p* < 0.01. (**c**) Alterations (mRNA upregulation/downregulation) of the *C1QBP* gene in COAD (TCGA PanCanAtlas; *n* = 439). Data was obtained using cBioPortal. (**d**) *C1QBP* mRNA expression was significantly associated with the copy number alteration status (ANOVA, *p* < 0.0001) in colon cancer. (****: *p* < 0.0001; *ns*: nonsignificant). (**e**) The survival curve comparing patients with high (red) and low (blue) expression in colon cancer was plotted from the PrognoScan database. Survival curve analysis was conducted using a threshold Cox *p*-value < 0.05. Abbreviations. COAD: colon adenocarcinoma; MACR: mucinous adenocarcinoma of the colon and rectum; RAD: rectal adenocarcinoma; CA: colorectal adenocarcinoma.

Colorectal cancer is the third most commonly diagnosed cancer in men and the second most common cancer in women [1]. Elevated *C1QBP* expression was observed in several colon cancer cell lines and patient tissues [45,47,48]. However, the correlation between *C1QBP* expression and colon cancer patient survival has not yet been investigated. Analysis of the Notterman dataset revealed a significantly higher expression of *C1QBP* in colon adenocarcinoma (COAD) patients compared to their normal counterparts (Figure 4a; Supplementary Table S5). In addition, analysis of the TCGA database through TCGA Wanderer and GEPIA showed a significantly higher expression of *C1QBP* in COAD patients than their normal counterparts (Figure 4b). Alterations in the *C1QBP* gene (TCGA PanCanAtlas dataset) were found in COAD, mucinous adenocarcinoma of the colon and rectum (MACR), rectal adenocarcinoma (RAD), and colorectal adenocarcinoma (CA) (Figure 4c). Upregulation of mRNA was found to be the most predominant alteration in all colon cancer subtypes (Figure 4c). There was a significant difference in *C1QBP* expression level between shallow deletions and diploid in the copy number alteration status in COAD, according to TCGA PanCanAtlas data-based analysis (Figure 4d). Analysis of GSE12945 dataset of PrognoScan database showed significantly higher patient survival in the low *C1QBP* expression group compared to the high *C1QBP* expression group (Figure 4e, Supplementary Table S6). These results suggest that colon cancers have significant *C1QBP* gene alterations related to augmented *C1QBP* expression, which are negatively correlated with overall survival in colon cancer patients. 

### 3.5. C1QBP Expression Pattern and Patient Survival in Bladder Cancer

**Figure 5:**
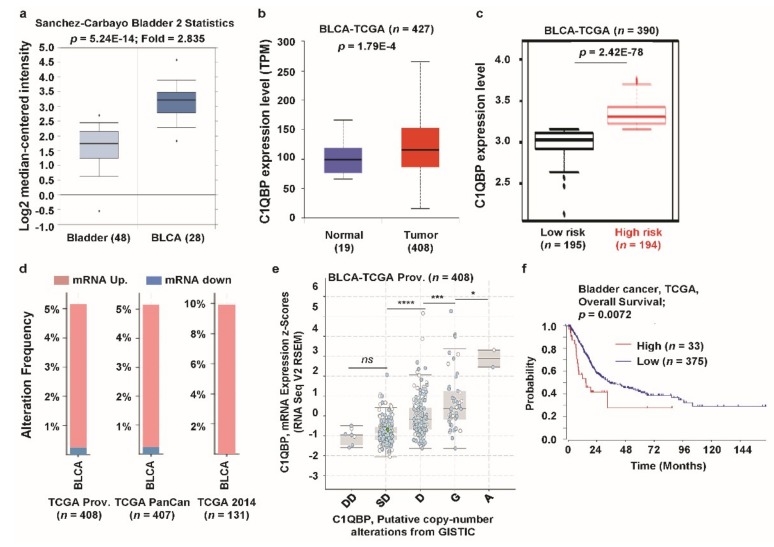
*C1QBP* expression pattern and patient survival analysis in bladder cancer, compared to *C1QBP* expression in normal tissue and cancer tissue. (**a**) The fold-change of *C1QBP* in bladder cancers was identified by our analyses, shown as a box plot. The box plot comparing specific *C1QBP* expression in normal (*n* = 48, left plot) and cancer tissue (*n* = 28, right plot) was derived from the Oncomine database. The analysis was shown in BLCA relative to in normal bladder. The asterisk above and below the box represent maximum and minimum value, respectively. (**b**) Expression of *C1QBP* gene in The Cancer Genome Atlas (TCGA) database. Box plots showing the *C1QBP* mRNA expression in BLCA tumor (red plot) and their normal (blue plot) tissues, using data from TCGA database through ULCAN. (**c**) *C1QBP* gene expression in BLCA patients from TCGA database. The box-plots generated using SurvExpress biomarker validation tool showing the gene expression in BLCA patients using cohorts from datasets generated by TCGA (*n* = 390). Box-plots show expression levels and *p*-values resulting from *t*-test of the difference expression between high-risk (red) and low-risk (green) groups in BLCA patients. (**d**) Alterations (mRNA upregulation/downregulation) of the *C1QBP* gene in BLCA (TCGA Prov., *n* = 408; TCGA PanCan atlas, *n* = 407; TCGA 2014, *n* = 131). Data were obtained using cBioPortal. (**e**) *C1QBP* mRNA expression was significantly associated with the copy number alteration status (ANOVA, *p* < 0.0001) in bladder cancer. (*: *p* < 0.05; ***: *p* < 0.001; ****: *p* < 0.0001; *ns*: nonsignificant). (**f**) The survival curve comparing patients with high (red) and low (blue) expression in bladder cancer was plotted from the R2: Genomics Analysis and Visualization Platform. Survival curve analysis was conducted using a threshold Cox *p*-value < 0.05.

*C1QBP* mRNA expression was highly upregulated in bladder cancer compared to the normal counterparts according to both the Oncomine and GENT databases (see Figure 1). From the detailed analysis, elevated expression of *C1QBP* was apparent in bladder urothelial carcinoma (BLCA) using the Oncomine (Figure 5a) and TCGA database (Figure 5b). Tissues from high-risk group of BLCA patients had significantly higher expression of *C1QBP* compared to that from the low-risk group in the analysis using SurvExpress biomarker validation tool (Figure 5c). We then focused whether alterations of *C1QBP* mRNA occurred in BLCA. Thus, we checked alteration frequencies of *C1QBP* mRNA in BLCA and the alterations were over 5% in TCGA Prov, TCGA PanCan, and TCGA 2014 datasets (Figure 5d). Upregulation of mRNA expression was the predominant alteration type in all datasets analyzed. The expression level of *C1QBP* mRNA was positively correlated with copy number alteration status from diploid and amplification (Figure 5e). Survival curve analysis with TCGA dataset showed that the high expression group had significantly poorer survival than the low-expression group (Figure 5f). These data suggested the elevated expression of *C1QBP* in bladder cancer was correlated with the cancer risk.

### 3.6. C1QBP Expression Pattern and Patient Survival in Lymphoma

**Figure 6:**
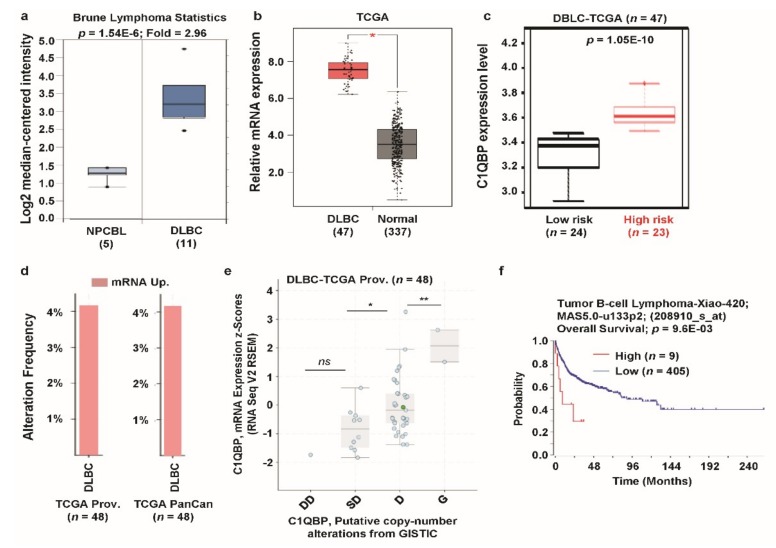
*C1QBP* expression pattern and patient survival analysis in lymphoma as compared to *C1QBP* expression in normal tissue and cancer tissue. (**a**) The fold-change of *C1QBP* in lymphoma was identified by our analyses, shown as a box plot. The box plot comparing specific *C1QBP* expression in normal (*n* = 5, left plot) and cancer tissue (*n* = 11, right plot) was derived from the Oncomine database. The analysis was shown in lymphoma relative to in normal tissue. The asterisk above and below the box represent maximum and minimum value, respectively. (**b**) Expression of the *C1QBP* gene in The Cancer Genome Atlas (TCGA) database. Box plots showing the *C1QBP* mRNA expression in DLBC (red plot) and their normal (blue plot) tissues, using data from the TCGA database through GEPIA. *: *p* < 0.01. (**c**) *C1QBP* gene expression in DLBC patients from the TCGA database. The box-plots generated using SurvExpress biomarker validation tool showing the gene expression in DLBC patients using cohorts from datasets generated by TCGA (*n* = 47). Box-plots show expression levels and *p*-values resulting from the *t*-test of the difference expression between high risk (red) and low risk (green) groups in DLBC patients. (**d**) Alterations (mRNA upregulation) of *C1QBP* gene in DLBC (TCGA Prov., *n* = 48; TCGA PanCan atlas, *n* = 48). Data was obtained using cBioPortal. (**e**) *C1QBP* mRNA expression was significantly associated with the copy number alteration status (ANOVA, *p* = 0.0009) in lymphoma. (*: *p* < 0.05; **: *p* < 0.01; ns: nonsignificant). (**f**) The survival curve comparing patients with high (red) and low (blue) expression in lymphoma was plotted from the R2: Genomics Analysis and Visualization Platform. Survival curve analysis was conducted using a threshold Cox *p*-value < 0.05.

*C1QBP* expression was greatly upregulated in lymphoma compared to the normal counterparts (see Figure 1a); thus, we elaborately analyzed the expression of *C1QBP* and its relevance in clinical outcomes in lymphoma datasets. In the Oncomine database, expression of *C1QBP* was enhanced approximately 3-fold in diffuse large B-cell lymphoma (DLBC) (Figure 6a). *C1QBP* expression was also significantly upregulated in DLBC according to TCGA database (Figure 6b). Among the DLBC patients, the high-risk group had a significantly higher expression of *C1QBP* than the lower risk group (Figure 6c). We then checked the alteration frequency of *C1QBP* genes in DLBC using TCGA data by cBioPortal. More than 4% alteration frequencies (*C1QBP* mRNA upregulation) were observed in DLBC in TCGA datasets (Figure 6d). In addition, mRNA expression of *C1QBP* is positively correlated with copy number alterations between shallow deletions and diploid and between diploid and gain in DLBC TCGA dataset. Next, we focused on the clinical outcomes of lymphoma patients with *C1QBP* expression. Lymphoma patients’ group with a high expression level of *C1QBP* mRNA showed significantly poor overall survival compared to the low expression group (Figure 6f). Overall, these data suggest the altered expression of *C1QBP* and its association of risk in lymphoma. 

### 3.7. C1QBP Expression Pattern and Patient Survival in Other Type of Cancers

Beside five types of cancers, we analyzed *C1QBP* expression and its relevance in clinical outcomes in other cancers. From the Oncomine analyses, *C1QBP* expression was found to be upregulated in various other cancers including brain, gastric, prostate, kidney, myeloma, and ovarian cancers (Supplementary Figure S1 and Table S7). TCGA database analyses also showed an upregulation in *C1QBP* expression in various other cancers including cholangio, glioblastoma multiforme, pancreatic, rectum, stomach, testicular germ cell, and thymoma cancers (Supplementary Figure S2). Moreover, survival analysis with *C1QBP* expression was performed using the R2: Kaplan Meier Scanner and the SurvExpress database. The analyses showed a negative correlation with patient survival in most of the investigated cancer types analyzed including pancreases, sarcoma, kidney, melanoma, myeloma, neuroblastoma, bladder, head and neck, and ovarian cancers (Supplementary Figure S3 and Table S8). These results suggest that *C1QBP* is probably involved in mechanisms that either aid in, or confer aggressiveness, in most cancers.

### 3.8. Differentially Expressed Genes with C1QBP Expression in Five Types of Cancers

**Figure 7:**
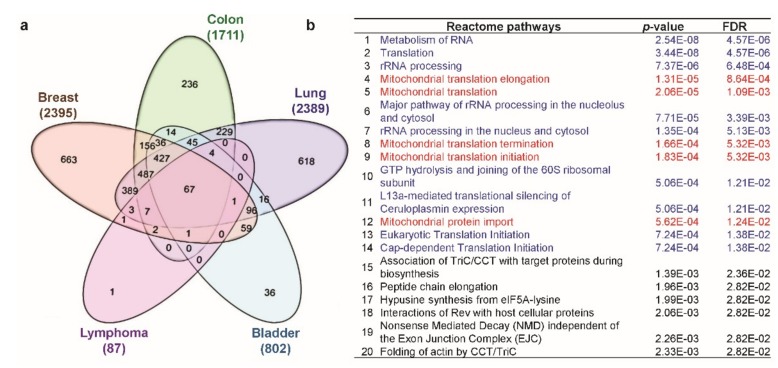
Analysis of positively correlated genes of *C1QBP* and their predicted pathway analysis using Reactome pathway analysis. (**a**) Venn diagram of genes positively correlated to *C1QBP*, showing coincident genes in breast, colon, lung, bladder, and lymphoma cancers. (**b**) Pathway analysis using Reactome pathway analysis. Gene ontology (GO) analysis using GOTermFinder functional annotation tool v1.0 is shown in Supplementary Table S9.

Finally, we aimed to find the potential signaling mechanism involved with *C1QBP* expression in cancers. To investigate the *C1QBP*-related pathways that might commonly play a role in various cancers, we analyzed transcriptome datasets from five different types of cancers, namely, breast, colon, lung, bladder cancers, and lymphoma using TCGA datasets through the R2: Genomics Analysis and Visualization Platform. Sixty-seven differently expressed genes (DEGs) were commonly upregulated with *C1QBP* in five selected cancers derived from the Venn diagram (Figure 7a), while only one DEG was commonly downregulated (Supplementary Figure S4). Total 67 common upregulated DEGs were classified using Reactome pathway analysis (Figure 7b) and the GOTermFinder functional annotation tool (Supplementary Table S9). The Reactome pathway analysis revealed that certain correlated genes were categorized in pathways related to the post-transcriptional control of gene expression including translation, such as metabolism of RNA, translation, and ribosomal RNA (rRNA) processing. Some categories were related to mitochondrial functions including mitochondrial translation, mitochondria protein import. Categories obtained from analysis using GOTermFinder functional annotation tool also contain terms related to RNA, translation, and mitochondria (Supplementary Table S9). As conveyed by these results, *C1QBP* could be associated with certain key pathways related to post-transcriptional control and mitochondrial function in cancer progression. 

## 4. Discussion

*C1QBP* is a multicompartmental protein that plays multiple roles in biological processes. Various roles of *C1QBP* have also been reported in multiple cancers. In this current study, we systematically analyzed *C1QBP* expression in various cancers by utilizing online expression databases and bioinformatics tools. Analysis of datasets revealed that *C1QBP* expression is significantly augmented in various cancer cells, compared to their normal counterparts. Level of *C1QBP* expression was positively correlated with copy number alterations and negatively correlated with patient survival in breast, lung, colon, and bladder cancers as well as lymphoma. These results strongly suggest the importance of *C1QBP* function in various cancer progression and prognosis. An association between patient survival and *C1QBP* expression was previously reported in breast [24,25], ovarian [26], endometrial [27], and cervical cancer [28]. In all previous studies, higher expression of *C1QBP* was negatively correlated with patient survival. Similarly, our analysis revealed that *C1QBP* expression is significantly higher in most types of cancer cells compared to their normal counterparts, and is negatively correlated with patient survival. This consistent trend in *C1QBP* expression in cancers suggests a commonality among different cancer types, where *C1QBP*-related molecular pathways may be functional. 

Differences of *C1QBP* mRNA expression between cancers and their counterparts were varied with cancer types. Among analyzed cancers in this study, the expression of *C1QBP* was more than the 2.5-fold augmented in bladder cancer and lymphoma compared to their normal counterparts (see Figure 5a and Figure 6a), which were bigger than other cancers. In these cancers, expression of *C1QBP* may play as a prognostic marker for the cancer progression which yet to be further validated by additional studies. Overexpressed *C1QBP* in cancers has been targeted by antibodies [49], A-tumor-homing peptide, LyP-1 [50], and small molecules [51] for therapeutic applications. Our study provides a systematic analysis suggesting *C1QBP* as a cancer target in various types of cancers.

Co-expression analysis shows that expression of *C1QBP* is positively correlated with gene expression categorized in RNA-related pathways and mitochondrial function. *C1QBP* knockout embryos have the severe defect of the mitochondrial respiratory chain and mitochondrial RNA-binding activity of *C1QBP* is well correlated with mitochondrial translation [52]. Knockdown of *C1QBP* reduces the cellular respiration of mitochondria, shifts metabolism from oxidative phosphorylation to glycolysis and suppresses tumor formation in vivo [53]. Increased expression of *C1QBP* promotes the oxidative phosphorylation in cancers [53]. Association of *C1QBP* in ribosome biogenesis by physical association of *C1QBP* with rRNA-processing factors has been previously reported [8,52]. However, our analysis is the first to reveal a positive correlation of *C1QBP* expression with a group of genes that are related to RNA-processing. Our findings may imply an association of *C1QBP* in post-transcriptional regulation of gene expression, especially for mitochondrial function. However, the roles of these groups in cancer progression remain open for further study.

## 5. Conclusions

In this systematic analysis of *C1QBP* expression in cancer databases, we provide evidence of the relationship between the altered expression of *C1QBP* and clinical outcomes. Our study uncovers the importance of *C1QBP* expression and possible *C1QBP*-related pathways in cancer progression. Therefore, our analysis may provide valuable insights into *C1QBP* as a potential therapeutic target for various human cancers.

## Supplementary Materials

The following are available online at https://www.mdpi.com/2077-0383/8/4/513/s1. Table S1: Datasets of *C1QBP* expression in breast cancer (Oncomine database); Table S2: Survival analyses of *C1QBP* in breast cancer (PrognoScan database); Table S3: Datasets of *C1QBP* expression in lung cancer (Oncomine database); Table S4: Survival analyses of *C1QBP* in lung cancer (PrognoScan database); Table S5: Datasets of *C1QBP* expression in colon cancer (Oncomine database); Table S6: Survival analyses of *C1QBP* in colon cancer (PrognoScan database); Table S7: Datasets of *C1QBP* expression in other cancers (Oncomine database); Table S8: Survival analyses of *C1QBP* in other cancers (PrognoScan database); Table S9: Gene ontology (GO) terms obtained from genes positively correlated with *C1QBP*; Figure S1: *C1QBP* expression analysis in different cancer types (Oncomine database); Figure S2: *C1QBP* expression analysis in different cancer types (TCGA database); Figure S3: Correlation of *C1QBP* gene expression with various cancer prognosis (R2: Kaplan Meier Scanner and SurvExpress); Figure S4: Analysis of negatively correlated genes of *C1QBP*.

## References

[1] Torre L.A., Bray F., Siegel R.L., Ferlay J., Lortet-Tieulent J., Jemal A. (2015). Global cancer statistics, 2012. CA Cancer J. Clin..

[2] Fitzmaurice C., Allen C., Barber R.M., Barregard L., Bhutta Z.A., Brenner H., Dicker D.J., Chimed-Orchir O., Dandona R., Global Burden of Disease Cancer Collaboration (2017). Global, Regional, and National Cancer Incidence, Mortality, Years of Life Lost, Years Lived With Disability, and Disability-Adjusted Life-years for 32 Cancer Groups, 1990 to 2015: A Systematic Analysis for the Global Burden of Disease Study. JAMA Oncol..

[3] Ghebrehiwet B., Lim B.L., Peerschke E.I., Willis A.C., Reid K.B. (1994). Isolation, cDNA cloning, and overexpression of a 33-kD cell surface glycoprotein that binds to the globular “heads” of C1q. J. Exp. Med..

[4] Gupta S., Batchu R.B., Datta K. (1991). Purification, partial characterization of rat kidney hyaluronic acid binding protein and its localization on the cell surface. Eur. J. Cell Biol..

[5] Soltys B.J., Kang D., Gupta R.S. (2000). Localization of P32 protein (gC1q-R) in mitochondria and at specific extramitochondrial locations in normal tissues. Histochem. Cell Biol..

[6] Krainer A.R., Mayeda A., Kozak D., Binns G. (1991). Functional expression of cloned human splicing factor SF2: Homology to RNA-binding proteins, U1 70K, and Drosophila splicing regulators. Cell.

[7] Majumdar M., Meenakshi J., Goswami S.K., Datta K. (2002). Hyaluronan binding protein 1 (HABP1)/C1QBP/p32 is an endogenous substrate for MAP kinase and is translocated to the nucleus upon mitogenic stimulation. Biochem. Biophys. Res. Commun..

[8] Feichtinger R.G., Olahova M., Kishita Y., Garone C., Kremer L.S., Yagi M., Uchiumi T., Jourdain A.A., Thompson K., D’Souza A.R. (2017). Biallelic C1QBP Mutations Cause Severe Neonatal-, Childhood-, or Later-Onset Cardiomyopathy Associated with Combined Respiratory-Chain Deficiencies. Am. J. Hum. Genet..

[9] Yoshikawa H., Komatsu W., Hayano T., Miura Y., Homma K., Izumikawa K., Ishikawa H., Miyazawa N., Tachikawa H., Yamauchi Y. (2011). Splicing factor 2-associated protein p32 participates in ribosome biogenesis by regulating the binding of Nop52 and fibrillarin to preribosome particles. Mol. Cell. Proteom..

[10] Petersen-Mahrt S.K., Estmer C., Ohrmalm C., Matthews D.A., Russell W.C., Akusjarvi G. (1999). The splicing factor-associated protein, p32, regulates RNA splicing by inhibiting ASF/SF2 RNA binding and phosphorylation. EMBO J..

[11] Leigh L.E., Ghebrehiwet B., Perera T.P., Bird I.N., Strong P., Kishore U., Reid K.B., Eggleton P. (1998). C1q-mediated chemotaxis by human neutrophils: Involvement of gClqR and G-protein signalling mechanisms. Biochem. J..

[12] Joseph K., Ghebrehiwet B., Peerschke E.I., Reid K.B., Kaplan A.P. (1996). Identification of the zinc-dependent endothelial cell binding protein for high molecular weight kininogen and factor XII: Identity with the receptor that binds to the globular “heads” of C1q (gC1q-R). Proc. Natl. Acad. Sci. USA.

[13] Herwald H., Dedio J., Kellner R., Loos M., Muller-Esterl W. (1996). Isolation and characterization of the kininogen-binding protein p33 from endothelial cells. Identity with the gC1q receptor. J. Biol. Chem..

[14] Pixley R.A., Espinola R.G., Ghebrehiwet B., Joseph K., Kao A., Bdeir K., Cines D.B., Colman R.W. (2011). Interaction of high-molecular-weight kininogen with endothelial cell binding proteins suPAR, gC1qR and cytokeratin 1 determined by surface plasmon resonance (BiaCore). Thromb. Haemost..

[15] Vegh Z., Kew R.R., Gruber B.L., Ghebrehiwet B. (2006). Chemotaxis of human monocyte-derived dendritic cells to complement component C1q is mediated by the receptors gC1qR and cC1qR. Mol. Immunol..

[16] Huang L., Chi J., Berry F.B., Footz T.K., Sharp M.W., Walter M.A. (2008). Human p32 is a novel FOXC1-interacting protein that regulates FOXC1 transcriptional activity in ocular cells. Investig. Ophthalmol. Vis. Sci..

[17] Chattopadhyay C., Hawke D., Kobayashi R., Maity S.N. (2004). Human p32, interacts with B subunit of the CCAAT-binding factor, CBF/NF-Y, and inhibits CBF-mediated transcription activation in vitro. Nucl. Acids Res..

[18] Matsumoto K., Kose S., Kuwahara I., Yoshimura M., Imamoto N., Yoshida M. (2018). Y-box protein-associated acidic protein (YBAP1/C1QBP) affects the localization and cytoplasmic functions of YB-1. Sci. Rep..

[19] Nguyen T., Ghebrehiwet B., Peerschke E.I. (2000). Staphylococcus aureus protein A recognizes platelet gC1qR/p33: A novel mechanism for staphylococcal interactions with platelets. Infect. Immun..

[20] Kittlesen D.J., Chianese-Bullock K.A., Yao Z.Q., Braciale T.J., Hahn Y.S. (2000). Interaction between complement receptor gC1qR and hepatitis C virus core protein inhibits T-lymphocyte proliferation. J. Clin. Investig..

[21] Waggoner S.N., Hall C.H., Hahn Y.S. (2007). HCV core protein interaction with gC1q receptor inhibits Th1 differentiation of CD4+ T cells via suppression of dendritic cell IL-12 production. J. Leukoc. Biol..

[22] Yadav G., Prasad R.L., Jha B.K., Rai V., Bhakuni V., Datta K. (2009). Evidence for inhibitory interaction of hyaluronan-binding protein 1 (HABP1/p32/gC1qR) with Streptococcus pneumoniae hyaluronidase. J. Biol. Chem..

[23] Xu L., Xiao N., Liu F., Ren H., Gu J. (2009). Inhibition of RIG-I and MDA5-dependent antiviral response by gC1qR at mitochondria. Proc. Natl. Acad. Sci. USA.

[24] Chen Y.B., Jiang C.T., Zhang G.Q., Wang J.S., Pang D. (2009). Increased expression of hyaluronic acid binding protein 1 is correlated with poor prognosis in patients with breast cancer. J. Surg. Oncol..

[25] Wang J., Song Y., Liu T., Shi Q., Zhong Z., Wei W., Huang S., Pang D. (2015). Elevated expression of HABP1 is a novel prognostic indicator in triple-negative breast cancers. Tumour Biol..

[26] Yu G., Wang J. (2013). Significance of hyaluronan binding protein (HABP1/P32/gC1qR) expression in advanced serous ovarian cancer patients. Exp. Mol. Pathol..

[27] Zhao J., Liu T., Yu G., Wang J. (2015). Overexpression of HABP1 correlated with clinicopathological characteristics and unfavorable prognosis in endometrial cancer. Tumour Biol..

[28] Zhang M., Li N., Liang Y., Liu J., Zhou Y., Liu C. (2017). Hyaluronic acid binding protein 1 overexpression is an indicator for disease-free survival in cervical cancer. Int. J. Clin. Oncol..

[29] Scully O.J., Yu Y., Salim A., Thike A.A., Yip G.W., Baeg G.H., Tan P.H., Matsumoto K., Bay B.H. (2015). Complement component 1, q subcomponent binding protein is a marker for proliferation in breast cancer. Exp. Biol. Med..

[30] Zhang X., Zhang F., Guo L., Wang Y., Zhang P., Wang R., Zhang N., Chen R. (2013). Interactome analysis reveals that C1QBP (complement component 1, q subcomponent binding protein) is associated with cancer cell chemotaxis and metastasis. Mol. Cell. Proteom..

[31] Yu H., Liu Q., Xin T., Xing L., Dong G., Jiang Q., Lv Y., Song X., Teng C., Huang D. (2013). Elevated expression of hyaluronic acid binding protein 1 (HABP1)/P32/C1QBP is a novel indicator for lymph node and peritoneal metastasis of epithelial ovarian cancer patients. Tumour Biol..

[32] Kim K.B., Yi J.S., Nguyen N., Lee J.H., Kwon Y.C., Ahn B.Y., Cho H., Kim Y.K., Yoo H.J., Lee J.S. (2011). Cell-surface receptor for complement component C1q (gC1qR) is a key regulator for lamellipodia formation and cancer metastasis. J. Biol. Chem..

[33] Wang Y., Fu D., Su J., Chen Y., Qi C., Sun Y., Niu Y., Zhang N., Yue D. (2017). C1QBP suppresses cell adhesion and metastasis of renal carcinoma cells. Sci. Rep..

[34] Rhodes D.R., Yu J., Shanker K., Deshpande N., Varambally R., Ghosh D., Barrette T., Pandey A., Chinnaiyan A.M. (2004). ONCOMINE: A cancer microarray database and integrated data-mining platform. Neoplasia.

[35] Rhodes D.R., Kalyana-Sundaram S., Mahavisno V., Varambally R., Yu J., Briggs B.B., Barrette T.R., Anstet M.J., Kincead-Beal C., Kulkarni P. (2007). Oncomine 3.0: Genes, pathways, and networks in a collection of 18,000 cancer gene expression profiles. Neoplasia.

[36] Shin G., Kang T.W., Yang S., Baek S.J., Jeong Y.S., Kim S.Y. (2011). GENT: Gene expression database of normal and tumor tissues. Cancer Inform..

[37] Chandrashekar D.S., Bashel B., Balasubramanya S.A.H., Creighton C.J., Ponce-Rodriguez I., Chakravarthi B., Varambally S. (2017). UALCAN: A Portal for Facilitating Tumor Subgroup Gene Expression and Survival Analyses. Neoplasia.

[38] Tang Z.F., Li C.W., Kang B.X., Gao G., Li C., Zhang Z.M. (2017). GEPIA: A web server for cancer and normal gene expression profiling and interactive analyses. Nucleic Acids Res..

[39] Diez-Villanueva A., Mallona I., Peinado M.A. (2015). Wanderer, an interactive viewer to explore DNA methylation and gene expression data in human cancer. Epigenetics Chromatin.

[40] Gao J., Aksoy B.A., Dogrusoz U., Dresdner G., Gross B., Sumer S.O., Sun Y., Jacobsen A., Sinha R., Larsson E. (2013). Integrative analysis of complex cancer genomics and clinical profiles using the cBioPortal. Sci. Signal..

[41] Cerami E., Gao J., Dogrusoz U., Gross B.E., Sumer S.O., Aksoy B.A., Jacobsen A., Byrne C.J., Heuer M.L., Larsson E. (2012). The cBio cancer genomics portal: An open platform for exploring multidimensional cancer genomics data. Cancer Discov..

[42] Aguirre-Gamboa R., Gomez-Rueda H., Martinez-Ledesma E., Martinez-Torteya A., Chacolla-Huaringa R., Rodriguez-Barrientos A., Tamez-Pena J.G., Trevino V. (2013). SurvExpress: An online biomarker validation tool and database for cancer gene expression data using survival analysis. PLoS ONE.

[43] Vastrik I., D’Eustachio P., Schmidt E., Gopinath G., Croft D., de Bono B., Gillespie M., Jassal B., Lewis S., Matthews L. (2007). Reactome: A knowledge base of biologic pathways and processes. Genome Biol..

[44] Boyle E.I., Weng S., Gollub J., Jin H., Botstein D., Cherry J.M., Sherlock G. (2004). GO::TermFinder–open source software for accessing Gene Ontology information and finding significantly enriched Gene Ontology terms associated with a list of genes. Bioinformatics.

[45] McGee A.M., Douglas D.L., Liang Y., Hyder S.M., Baines C.P. (2011). The mitochondrial protein C1qbp promotes cell proliferation, migration and resistance to cell death. Cell Cycle.

[46] Li W., Zhang X., Wang W., Sun R., Liu B., Ma Y., Zhang W., Ma L., Jin Y., Yang S. (2017). Quantitative proteomics analysis of mitochondrial proteins in lung adenocarcinomas and normal lung tissue using iTRAQ and tandem mass spectrometry. Am. J. Transl. Res..

[47] Kim K., Kim M.J., Kim K.H., Ahn S.A., Kim J.H., Cho J.Y., Yeo S.G. (2017). C1QBP is upregulated in colon cancer and binds to apolipoprotein A-I. Exp. Ther. Med..

[48] Dembitzer F.R., Kinoshita Y., Burstein D., Phelps R.G., Beasley M.B., Garcia R., Harpaz N., Jaffer S., Thung S.N., Unger P.D. (2012). gC1qR expression in normal and pathologic human tissues: Differential expression in tissues of epithelial and mesenchymal origin. J. Histochem. Cytochem..

[49] Sanchez-Martin D., Cuesta A.M., Fogal V., Ruoslahti E., Alvarez-Vallina L. (2011). The multicompartmental p32/gClqR as a new target for antibody-based tumor targeting strategies. J. Biol. Chem..

[50] Li X., Jin Q., Chen T., Zhang B., Zheng R., Wang Z., Zheng H. (2009). LyP-1 ultrasonic microbubbles targeting to cancer cell as tumor bio-acoustics markers or drug carriers: Targeting efficiency evaluation in, microfluidic channels. Conf. Proc. IEEE Eng. Med. Biol. Soc..

[51] Yenugonda V., Nomura N., Kouznetsova V., Tsigelny I., Fogal V., Nurmemmedov E., Kesari S., Babic I. (2017). A novel small molecule inhibitor of p32 mitochondrial protein overexpressed in glioma. J. Transl. Med..

[52] Yagi M., Uchiumi T., Takazaki S., Okuno B., Nomura M., Yoshida S., Kanki T., Kang D. (2012). p32/gC1qR is indispensable for fetal development and mitochondrial translation: Importance of its RNA-binding ability. Nucl. Acids Res..

[53] Fogal V., Richardson A.D., Karmali P.P., Scheffler I.E., Smith J.W., Ruoslahti E. (2010). Mitochondrial p32 protein is a critical regulator of tumor metabolism via maintenance of oxidative phosphorylation. Mol. Cell. Biol..

