# Supplementary material for: Systematic Multiomics Analysis of Alterations in C1QBP mRNA Expression and Relevance for Clinical Outcomes in Cancers
Source: J Clin Med. 2019 Apr 15;8(4):513. doi: 10.3390/jcm8040513 (PMC6517981; doi:10.3390/jcm8040513)
Supplement: Supplementary file 1 [file jcm-08-00513-s001.pdf]

Supplementary information: Systematic Multiomics Analysis of *C1QBP* mRNA Expression Alterations and Its Relevance of Clinical Outcomes in Cancers

Subbroto Kumar Saha[1], Kyung Eun Kim[2], S.M. Riazul Islam[3], Ssang-Goo Cho[1,*] and Minchan Gil[1,*]

[1] Department of Stem Cell and Regenerative Biotechnology, Incurable Disease Animal Model & Stem Cell Institute (IDASI), Konkuk University, 120 Neungdong-ro, Gwangjin-gu, Seoul, 05029 Republic of Korea; subbroto@konkuk.ac.kr (S.K.S.), ssangoo@konkuk.ac.kr (S-G. C), minchangil@gmail.com (M.G.)

[2] Department of Cosmetic Sciences, Sookmyung Women's University, Cheongpa-ro 47-gil 100 (Cheongpa-dong 2ga), Yongsan-gu, Seoul 04310, Republic of Korea; kyungeun@sookmyung.ac.kr

[3] Department of Computer Science and Engineering, Sejong University, 209, Neungdong-ro, Gwangjin-gu, Seoul, 05006, Republic of Korea; riaz@sejong.ac.kr (S.M.R.I.)

* Correspondence: ssangoo@konkuk.ac.kr; minchangil@gmail.com; Tel.: +82-10-2773-0173 (M.G.) +82-2-450-4207 (S.-G.C.); Fax.: +82-2-444-4207 (S.-G.C. and M.G.)

Supplementary Table S1. Datasets of *C1QBP* expression in breast cancer (Oncomine database).

Dataset	Normal (Cases)	Tumor (Cases)	Fold change	t-Test	*p*-value	Rank (%)
Curtis	Breast (144)	Tubular Breast Carcinoma (67)	1.45	9.507	2.82E-16	8
	Breast (144)	Breast Carcinoma (14)	1.585	4.275	3.94E-04	9
	Breast (144)	Invasive Breast Carcinoma (21)	1.382	4.217	1.67E-04	11
	Breast (144)	Invasive Ductal Breast Carcinoma (1,556)	1.359	14.336	9.46E-34	15
	Breast (144)	Invasive Ductal and Invasive Lobular Breast Carcinoma (90)	1.326	7.262	1.06E-11	15
	Breast (144)	Ductal Breast Carcinoma in Situ (10)	1.393	3.215	5.00E-03	15
	Breast (144)	Mucinous Breast Carcinoma (46)	1.399	5.779	1.70E-07	16
	Breast (144)	Medullary Breast Carcinoma (32)	1.445	4.28	7.12E-05	18
Turashvili	Ductal Breast Cell (10)	Invasive Ductal Breast Carcinoma (5)	-1.584	-2.25	0.022	7
Finak	Breast (6)	Invasive Breast Carcinoma (53)	-2.716	-9.253	1.20E-11	15

Supplementary Table S2. Survival analyses of *C1QBP* in breast cancer (PrognoScan database).

DATASET	ENDPOINT	ARRAY TYPE	PROBE ID	N	COX *p*-VALUE	HR [95% CI-low CI-upp]
GSE19615	Distant Metastasis Free Survival	HG-U133_Plus_2	208910_s_at	115	0.371956	0.52 [0.12 - 2.20]
GSE19615	Distant Metastasis Free Survival	HG-U133_Plus_2	214214_s_at	115	0.169939	0.35 [0.08 - 1.57]
GSE12276	Relapse Free Survival	HG-U133_Plus_2	214214_s_at	204	0.43914	1.14 [0.82 - 1.59]
GSE12276	Relapse Free Survival	HG-U133_Plus_2	208910_s_at	204	0.537201	1.10 [0.81 - 1.50]
GSE6532-GPL570	Relapse Free Survival	HG-U133_Plus_2	214214_s_at	87	0.565589	1.24 [0.59 - 2.62]
GSE6532-GPL570	Relapse Free Survival	HG-U133_Plus_2	208910_s_at	87	0.462749	1.30 [0.65 - 2.60]
GSE6532-GPL570	Distant Metastasis Free Survival	HG-U133_Plus_2	214214_s_at	87	0.565589	1.24 [0.59 - 2.62]
GSE6532-GPL570	Distant Metastasis Free Survival	HG-U133_Plus_2	208910_s_at	87	0.462749	1.30 [0.65 - 2.60]
GSE9195	Relapse Free Survival	HG-U133_Plus_2	214214_s_at	77	0.439715	0.68 [0.25 - 1.83]
GSE9195	Relapse Free Survival	HG-U133_Plus_2	208910_s_at	77	0.314056	0.66 [0.29 - 1.49]
GSE9195	Distant Metastasis Free Survival	HG-U133_Plus_2	208910_s_at	77	0.566883	0.76 [0.30 - 1.94]
GSE9195	Distant Metastasis Free Survival	HG-U133_Plus_2	214214_s_at	77	0.746708	0.83 [0.27 - 2.58]
GSE12093	Distant Metastasis Free Survival	HG-U133A	208910_s_at	136	0.468174	0.77 [0.38 - 1.57]
GSE12093	Distant Metastasis Free Survival	HG-U133A	214214_s_at	136	0.423397	1.50 [0.55 - 4.07]
GSE11121	Distant Metastasis Free Survival	HG-U133A	208910_s_at	200	0.578022	0.80 [0.36 - 1.77]
GSE11121	Distant Metastasis Free Survival	HG-U133A	214214_s_at	200	0.063933	2.07 [0.96 - 4.49]
GSE9893	Overall Survival	MLRG Human 21K V12.0	19328	155	0.011484	1.32 [1.06 - 1.64]
GSE2034	Distant Metastasis Free Survival	HG-U133A	208910_s_at	286	0.354935	0.81 [0.51 - 1.27]
GSE2034	Distant Metastasis Free Survival	HG-U133A	214214_s_at	286	0.878539	1.04 [0.64 - 1.68]
GSE1456-GPL96	Overall Survival	HG-U133A	208910_s_at	159	0.207785	1.49 [0.80 - 2.79]
GSE1456-GPL96	Disease Specific Survival	HG-U133A	214214_s_at	159	0.155031	1.85 [0.79 - 4.34]
GSE1456-GPL96	Relapse Free Survival	HG-U133A	208910_s_at	159	0.110369	1.66 [0.89 - 3.11]
GSE1456-GPL96	Overall Survival	HG-U133A	214214_s_at	159	0.28609	1.49 [0.72 - 3.12]

GSE1456-GPL96	Disease Specific Survival	HG-U133A	208910_s_at	159	0.221318	1.58 [0.76 - 3.29]
GSE1456-GPL96	Relapse Free Survival	HG-U133A	214214_s_at	159	0.080311	1.90 [0.93 - 3.90]
GSE7378	Disease Free Survival	U133AAofAv2	208910_s_at	54	0.275125	1.44 [0.75 - 2.79]
GSE7378	Disease Free Survival	U133AAofAv2	214214_s_at	54	0.254049	1.62 [0.71 - 3.72]
E-TABM-158	Distant Metastasis Free Survival	HG-U133A	208910_s_at	117	0.406336	0.86 [0.59 - 1.23]
E-TABM-158	Overall Survival	HG-U133A	208910_s_at	117	0.6718	0.94 [0.69 - 1.27]
E-TABM-158	Relapse Free Survival	HG-U133A	214214_s_at	117	0.73798	0.94 [0.66 - 1.35]
E-TABM-158	Disease Specific Survival	HG-U133A	214214_s_at	117	0.489297	0.87 [0.58 - 1.30]
E-TABM-158	Overall Survival	HG-U133A	214214_s_at	117	0.73798	0.94 [0.66 - 1.35]
E-TABM-158	Distant Metastasis Free Survival	HG-U133A	214214_s_at	117	0.668049	0.91 [0.59 - 1.41]
E-TABM-158	Relapse Free Survival	HG-U133A	208910_s_at	117	0.6718	0.94 [0.69 - 1.27]
E-TABM-158	Disease Specific Survival	HG-U133A	208910_s_at	117	0.377166	0.86 [0.61 - 1.21]
GSE3494-GPL96	Disease Specific Survival	HG-U133A	208910_s_at	236	0.477552	1.22 [0.70 - 2.12]
GSE3494-GPL96	Disease Specific Survival	HG-U133A	214214_s_at	236	0.494525	1.25 [0.66 - 2.38]
GSE4922-GPL96	Disease Free Survival	HG-U133A	214214_s_at	249	0.174824	1.39 [0.86 - 2.25]
GSE4922-GPL96	Disease Free Survival	HG-U133A	208910_s_at	249	0.151466	1.37 [0.89 - 2.09]
GSE2990	Distant Metastasis Free Survival	HG-U133A	208910_s_at	125	0.310531	1.28 [0.79 - 2.06]
GSE2990	Relapse Free Survival	HG-U133A	214214_s_at	62	0.502492	0.78 [0.37 - 1.63]
GSE2990	Relapse Free Survival	HG-U133A	208910_s_at	125	0.536003	1.12 [0.78 - 1.63]
GSE2990	Distant Metastasis Free Survival	HG-U133A	214214_s_at	125	0.169222	1.54 [0.83 - 2.87]
GSE2990	Distant Metastasis Free Survival	HG-U133A	208910_s_at	54	0.638987	0.82 [0.35 - 1.90]
GSE2990	Relapse Free Survival	HG-U133A	214214_s_at	125	0.307875	1.28 [0.80 - 2.06]
GSE2990	Distant Metastasis Free Survival	HG-U133A	214214_s_at	54	0.457043	0.73 [0.31 - 1.69]
GSE2990	Relapse Free Survival	HG-U133A	208910_s_at	62	0.784708	0.90 [0.44 - 1.86]
GSE7390	Distant Metastasis Free Survival	HG-U133A	214214_s_at	198	0.618647	1.11 [0.74 - 1.65]
GSE7390	Overall Survival	HG-U133A	214214_s_at	198	0.624726	1.11 [0.73 - 1.69]

GSE7390	Relapse Free Survival	HG-U133A	208910_s_at	198	0.547065	1.10 [0.80 - 1.52]
GSE7390	Distant Metastasis Free Survival	HG-U133A	208910_s_at	198	0.775386	1.06 [0.72 - 1.55]
GSE7390	Relapse Free Survival	HG-U133A	214214_s_at	198	0.444162	1.14 [0.81 - 1.60]
GSE7390	Overall Survival	HG-U133A	208910_s_at	198	0.818554	1.05 [0.70 - 1.57]

Supplementary Table S3. Datasets of *C1QBP* expression in lung cancer (Oncomine database).

Dataset	Normal (Cases)	Tumor (Cases)	Fold change	t-Test	*p*-value	Rank (%)
Wachi	Lung (5)	Squamous Cell Lung Carcinoma (5)	1.838	4.106	0.003	6
Hou	Lung (65)	Large Cell Lung Carcinoma (19)	1.928	5.892	4.39E-06	6
	Lung (65)	Squamous Cell Lung Carcinoma (27)	1.867	7.139	2.32E-08	8
	Lung (65)	Lung Adenocarcinoma (45)	1.363	4.857	4.16E-06	13
Landi	Lung (49)	Lung Adenocarcinoma (58)	1.468	5.59	9.42E-08	12
Su	Lung (30)	Lung Adenocarcinoma (27)	1.235	2.037	0.024	23
Okayama	Lung (20)	Lung Adenocarcinoma (226)	1.182	3.143	0.002	33

Supplementary Table S4. Survival analyses of *C1QBP* in lung cancer (PrognoScan database).

DATASET	SUBTYPE	ENDPOINT	ARRAY TYPE	PROBE ID	N	COX *p*-VALUE	HR [95% CI-low CI-upp]
jacob-00182-CANDF	Adenocarcinoma	Overall Survival	HG-U133A	208910_s_at	82	0.75218	1.10 [0.61 - 1.98]
jacob-00182-CANDF	Adenocarcinoma	Overall Survival	HG-U133A	214214_s_at	82	0.61874	1.21 [0.57 - 2.59]
jacob-00182-HLM	Adenocarcinoma	Overall Survival	HG-U133A	208910_s_at	79	0.08171	1.60 [0.94 - 2.71]
jacob-00182-HLM	Adenocarcinoma	Overall Survival	HG-U133A	214214_s_at	79	0.04994	1.77 [1.00 - 3.13]
jacob-00182-MSK	Adenocarcinoma	Overall Survival	HG-U133A	214214_s_at	104	0.41293	0.74 [0.36 - 1.53]
jacob-00182-MSK	Adenocarcinoma	Overall Survival	HG-U133A	208910_s_at	104	0.40605	0.80 [0.48 - 1.35]
GSE13213	Adenocarcinoma	Overall Survival	G4112F	A_23_P370434	117	0.08626	1.65 [0.93 - 2.91]
GSE31210	Adenocarcinoma	Overall Survival	HG-U133_Plus_2	208910_s_at	204	0.45502	0.65 [0.21 - 2.03]
GSE31210	Adenocarcinoma	Overall Survival	HG-U133_Plus_2	214214_s_at	204	0.64079	0.74 [0.21 - 2.63]
GSE31210	Adenocarcinoma	Relapse Free Survival	HG-U133_Plus_2	214214_s_at	204	0.36443	1.52 [0.62 - 3.73]
GSE31210	Adenocarcinoma	Relapse Free Survival	HG-U133_Plus_2	208910_s_at	204	0.40748	1.41 [0.63 - 3.18]
jacob-00182-UM	Adenocarcinoma	Overall Survival	HG-U133A	208910_s_at	178	0.90606	0.97 [0.64 - 1.48]
jacob-00182-UM	Adenocarcinoma	Overall Survival	HG-U133A	214214_s_at	178	0.69053	1.10 [0.69 - 1.75]
GSE11117	NSCLC	Overall Survival	Novachip human 34.5k	H200006536	41	0.82862	0.93 [0.49 - 1.78]
GSE3141	NSCLC	Overall Survival	HG-U133_Plus_2	208910_s_at	111	0.36701	0.82 [0.54 - 1.26]
GSE3141	NSCLC	Overall Survival	HG-U133_Plus_2	214214_s_at	111	0.99807	1.00 [0.66 - 1.51]
GSE14814	NSCLC	Overall Survival	HG-U133A	208910_s_at	90	0.43197	1.18 [0.78 - 1.81]
GSE14814	NSCLC	Disease Specific Survival	HG-U133A	208910_s_at	90	0.41344	1.22 [0.76 - 1.97]
GSE14814	NSCLC	Disease Specific Survival	HG-U133A	214214_s_at	90	0.55358	1.13 [0.75 - 1.70]

GSE14814	NSCLC	Overall Survival	HG-U133A	214214_s_at	90	0.44367	1.15 [0.80 - 1.65]
GSE8894	NSCLC	Relapse Free Survival	HG-U133_Plus_2	214214_s_at	138	0.99951	1.00 [0.76 - 1.31]
GSE8894	NSCLC	Relapse Free Survival	HG-U133_Plus_2	208910_s_at	138	0.91912	1.01 [0.81 - 1.26]
GSE4573	Squamous cell carcinoma	Overall Survival	HG-U133A	214214_s_at	129	0.48793	1.22 [0.69 - 2.17]
GSE4573	Squamous cell carcinoma	Overall Survival	HG-U133A	208910_s_at	129	0.47779	1.24 [0.68 - 2.27]
GSE17710	Squamous cell carcinoma	Relapse Free Survival	Agilent-UNC-custom-4X44K	16225	56	0.67896	0.85 [0.40 - 1.83]
GSE17710	Squamous cell carcinoma	Overall Survival	Agilent-UNC-custom-4X44K	16225	56	0.78275	0.89 [0.40 - 1.99]

Supplementary Table S5. Datasets of *C1QBP* expression in colon cancer (Oncomine database).

Dataset	Normal (Cases)	Tumor (Cases)	Fold change	t-Test	*p*-value	Rank (%)
Sabates-Bellver	Ascending Colon (4)	Colon Adenoma (25)	1.99	10.188	2.16E-12	4
	Ascending Colon (4)	Rectal Adenoma (7)	2.386	7.661	6.33E-05	10
Notterman	Colon (18)	Colon Adenocarcinoma (18)	2.499	2.754	0.005	10
Graudens	Colon (12)	Colorectal Carcinoma (18)	1.133	2.123	0.022	22
Skrzypczak	Colorectal Tissue (24)	Colorectal Adenocarcinoma (45)	1.588	5.692	7.11E-07	9
	Colorectal Tissue (24)	Colorectal Carcinoma (36)	1.502	4.197	6.68E-05	14
TCGA	Colon (19)	Rectosigmoid Adenocarcinoma (3)	1.258	3.791	3.00E-03	10
	Colon (19)	Colon Mucinous Adenocarcinoma (22)	1.601	5.051	8.81E-06	15
	Colon (19)	Colon Adenocarcinoma (101)	1.307	4.634	1.00E-05	24
	Colon (19)	Cecum Adenocarcinoma (22)	1.359	3.599	5.04E-04	25
Skrzypczak 2	Colon (10)	Colon Adenoma (5)	1.896	3.612	2.00E-03	17
	Colon (10)	Colon Carcinoma (5)	1.424	3.803	2.00E-03	21
Hong	Colon (12)	Colorectal Carcinoma (70)	1.344	2.834	5.00E-03	31
Kaiser	Colon (5)	Colon Mucinous Adenocarcinoma (13)	1.225	1.932	3.60E-02	42
Gaedcke	Rectum (65)	Rectal Adenocarcinoma (65)	1.117	2.342	1.00E-02	44

Supplementary Table S6. Survival analyses of *C1QBP* in colon cancer (PrognoScan database).

DATASET	CANCER TYPE	ENDPOINT	ARRAY TYPE	PROBE ID	N	COX *p*-VALUE	HR [95% CI-low CI-upp]
GSE12945	Colorectal cancer	Disease Free Survival	HG-U133A	208910_s_at	51	0.551898	0.76 [0.30 - 1.89]
GSE12945	Colorectal cancer	Overall Survival	HG-U133A	208910_s_at	62	0.0878153	1.69 [0.93 - 3.08]
GSE12945	Colorectal cancer	Disease Free Survival	HG-U133A	214214_s_at	51	0.563635	0.75 [0.29 - 1.96]
GSE12945	Colorectal cancer	Overall Survival	HG-U133A	214214_s_at	62	0.0736583	1.96 [0.94 - 4.09]
GSE17536	Colorectal cancer	Disease Specific Survival	HG-U133_Plus_2	208910_s_at	177	0.611809	0.87 [0.52 - 1.47]
GSE17536	Colorectal cancer	Overall Survival	HG-U133_Plus_2	214214_s_at	177	0.776329	1.07 [0.68 - 1.69]
GSE17536	Colorectal cancer	Overall Survival	HG-U133_Plus_2	208910_s_at	177	0.772939	1.07 [0.68 - 1.68]
GSE17536	Colorectal cancer	Disease Free Survival	HG-U133_Plus_2	214214_s_at	145	0.573441	0.83 [0.44 - 1.58]
GSE17536	Colorectal cancer	Disease Free Survival	HG-U133_Plus_2	208910_s_at	145	0.627296	0.86 [0.46 - 1.60]
GSE17536	Colorectal cancer	Disease Specific Survival	HG-U133_Plus_2	214214_s_at	177	0.579844	0.86 [0.51 - 1.46]
GSE14333	Colorectal cancer	Disease Free Survival	HG-U133_Plus_2	214214_s_at	226	0.0405743	0.54 [0.30 - 0.97]
GSE14333	Colorectal cancer	Disease Free Survival	HG-U133_Plus_2	208910_s_at	226	0.0805072	0.64 [0.39 - 1.06]
GSE17537	Colorectal cancer	Disease Free Survival	HG-U133_Plus_2	208910_s_at	55	0.9651	0.99 [0.54 - 1.81]
GSE17537	Colorectal cancer	Disease Free Survival	HG-U133_Plus_2	214214_s_at	55	0.89308	1.05 [0.52 - 2.11]
GSE17537	Colorectal cancer	Disease Specific Survival	HG-U133_Plus_2	214214_s_at	49	0.697074	1.21 [0.47 - 3.11]
GSE17537	Colorectal cancer	Disease Specific Survival	HG-U133_Plus_2	208910_s_at	49	0.874998	0.94 [0.42 - 2.11]
GSE17537	Colorectal cancer	Overall Survival	HG-U133_Plus_2	214214_s_at	55	0.540262	1.24 [0.63 - 2.44]
GSE17537	Colorectal cancer	Overall Survival	HG-U133_Plus_2	208910_s_at	55	0.647753	1.15 [0.64 - 2.05]

Supplementary Table S7. Datasets of *C1QBP* expression in other cancers (Oncomine database).

Cancer type	Dataset	Normal (Cases)	Tumor (Cases)	Fold change	t-Test	*p*-value	Rank (%)
Bladder	Sanchez-Carbayo	Bladder (48)	Superficial Bladder Cancer (28)	2.835	9.418	5.24E-14	6
	Dyrskjot 3	Bladder (9)	Superficial Bladder Cancer (28)	2.421	5.162	1.50E-05	13
		Bladder (9)	Infiltrating Bladder Urothelial Carcinoma (13)	2.81	4.053	2.49E-04	14
Brain	French	Brain (6)	Anaplastic Oligoastrocytoma (4)	2.104	4.313	2.00E-03	5
		Brain (6)	Anaplastic Oligodendroglioma (23)	2.076	4.337	1.00E-03	16
	TCGA	Brain (10)	Brain Glioblastoma (542)	1.408	4.769	3.82E-04	30
	Lee	Neural Stem Cell (3)	Glioblastoma (22)	-2.261	-4.541	1.20E-02	43
Cervix	Biewenga	Cervix Uteri (5)	Cervical Squamous Cell Carcinoma (40)	1.639	6.228	6.14E-05	13
Esophagus	Su Esophagus 2	Esophagus (53)	Esophageal Squamous Cell Carcinoma (53)	1.669	6.25	4.84E-09	10
	Kim	Esophagus (28)	Esophageal Adenocarcinoma (75)	-2.184	-6.514	2.14E-09	12
		Esophagus (28)	Barrett's Esophagus (15)	-1.529	-3.142	3.00E-03	32
Gastric	DErrico	Gastric Mucosa (31)	Gastric Intestinal Type Adenocarcinoma (26)	2.125	6.149	5.55E-08	8
		Gastric Mucosa (31)	Diffuse Gastric Adenocarcinoma (6)	2.153	3.328	6.00E-03	11
		Gastric Mucosa (31)	Gastric Mixed Adenocarcinoma (4)	2.153	3.957	5.00E-03	15
	Cho	Gastric Tissue (19)	Gastric Mixed Adenocarcinoma (10)	1.708	4.546	6.46E-05	2
		Gastric Tissue (19)	Diffuse Gastric Adenocarcinoma (31)	1.455	3.185	1.00E-03	12
	Wang	Gastric Mucosa (12)	Gastric Cancer (12)	1.526	3.103	3.00E-03	10
Head-Neck	Sengupta	Nasopharynx (10)	Nasopharyngeal Carcinoma (31)	2.026	5.209	7.55E-06	4
	Giordano	Thyroid Gland (4)	Thyroid Gland Oncocytic Follicular Carcinoma (8)	1.213	4.665	4.55E-04	2
		Thyroid Gland (4)	Thyroid Gland Oncocytic Adenoma (7)	1.292	4.266	2.00E-03	7
Kidney	Yusenko	Fetal Kidney (2)	Papillary Renal Cell Carcinoma (19)	2.059	7.493	5.37E-07	1
		Fetal Kidney (2)	Renal Oncocytoma (4)	1.88	8.146	4.35E-05	2
		Fetal Kidney (2)	Chromophobe Renal Cell Carcinoma (4)	-1.639	-3.515	8.00E-03	7
	Jones	Kidney (23)	Papillary Renal Cell Carcinoma (11)	2.026	5.41	2.16E-05	15
		Kidney (23)	Renal Pelvis Urothelial Carcinoma (8)	2.231	3.866	2.00E-03	22
		Kidney (23)	Renal Oncocytoma (12)	1.396	2.427	1.30E-02	36

	Beroukhim	Renal Cortex (10)	Non-Hereditary Clear Cell Renal Cell Carcinoma (27)	-1.332	-2.962	3.00E-03	20
	Lenburg	Kidney (9)	Clear Cell Renal Cell Carcinoma (9)	-1.792	-2.586	1.00E-02	15
Lymphoma	Brune	Memory B-Lymphocyte (5)	Burkitt's Lymphoma (5)	5.161	8.914	4.58E-06	3
		Memory B-Lymphocyte (5)	Diffuse Large B-Cell Lymphoma (11)	2.96	6.153	1.54E-06	3
	Eckerle	Natural Killer Cell (5)	Anaplastic Large Cell Lymphoma, ALK-Positive (5)	3.869	6.994	1.83E-04	6
Myeloma	Agnelli	Plasma Cell (5)	Plasma Cell Leukemia (9)	2.549	3.889	0.001	8
	Zhan	Bone Marrow (22)	Smoldering Myeloma (12)	2.662	6.893	6.04E-07	6
Parathyroid	Morrison	Parathyroid Gland (5)	Parathyroid Hyperplasia (8)	3.145	4.069	9.53E-04	3
		Parathyroid Gland (5)	Non-Familial Multiple Gland Neoplasia (10)	3.373	4.209	5.54E-04	4
		Parathyroid Gland (5)	Familial Parathyroid Hyperplasia (3)	2.965	3.556	0.011	4
		Parathyroid Gland (5)	Parathyroid Gland Adenoma (35)	4.232	7.217	2.17E-04	5
Seminoma	Korkola	Testis (6)	Seminoma, NOS (12)	2.592	6.924	4.03E-06	10

Supplementary Table S8. Survival analyses of *C1QBP* in other cancers (PrognoScan database).

DATASET	CANCER TYPE	SUBTYPE	ENDPOINT	ARRAY TYPE	PROBE ID	N	COX *p*-VALUE	HR [95% CI-low CI-upp]
GSE5287	Bladder cancer		Overall Survival	HG-U133A	214214_s_at	30	0.062814	0.55 [0.29 - 1.03]
GSE5287	Bladder cancer		Overall Survival	HG-U133A	208910_s_at	30	0.281045	0.73 [0.41 - 1.30]
GSE13507	Bladder cancer		Overall Survival	Human-6 v2	ILMN_1668996	165	0.755308	1.05 [0.77 - 1.42]
GSE13507	Bladder cancer		Disease Specific Survival	Human-6 v2	ILMN_1668996	165	0.93875	1.02 [0.64 - 1.61]
GSE12417-GPL96	Blood cancer	AML	Overall Survival	HG-U133A	208910_s_at	163	0.469962	1.10 [0.85 - 1.40]
GSE12417-GPL96	Blood cancer	AML	Overall Survival	HG-U133A	214214_s_at	163	0.453089	1.10 [0.85 - 1.43]
GSE12417-GPL570	Blood cancer	AML	Overall Survival	HG-U133_Plus_2	214214_s_at	79	0.105577	1.48 [0.92 - 2.38]
GSE12417-GPL570	Blood cancer	AML	Overall Survival	HG-U133_Plus_2	208910_s_at	79	0.127272	1.35 [0.92 - 2.00]
GSE5122	Blood cancer	AML	Overall Survival	HG-U133A	214214_s_at	58	0.329452	0.76 [0.44 - 1.32]
GSE5122	Blood cancer	AML	Overall Survival	HG-U133A	208910_s_at	58	0.56243	0.90 [0.63 - 1.29]
GSE8970	Blood cancer	AML	Overall Survival	HG-U133A	208910_s_at	34	0.286225	1.54 [0.69 - 3.43]
GSE8970	Blood cancer	AML	Overall Survival	HG-U133A	214214_s_at	34	0.571717	1.21 [0.63 - 2.33]
GSE4475	Blood cancer	B-cell lymphoma	Overall Survival	HG-U133A	214214_s_at	158	0.0946979	0.78 [0.58 - 1.04]
GSE4475	Blood cancer	B-cell lymphoma	Overall Survival	HG-U133A	208910_s_at	158	0.0295261	0.71 [0.53 - 0.97]
E-TABM-346	Blood cancer	DLBCL	Overall Survival	HG-U133A	208910_s_at	53	0.134125	0.55 [0.25 - 1.21]
E-TABM-346	Blood cancer	DLBCL	Overall Survival	HG-U133A	214214_s_at	53	0.169011	0.54 [0.23 - 1.30]
E-TABM-346	Blood cancer	DLBCL	Event Free Survival	HG-U133A	208910_s_at	53	0.182424	0.59 [0.27 - 1.28]
E-TABM-346	Blood cancer	DLBCL	Event Free Survival	HG-U133A	214214_s_at	53	0.141018	0.53 [0.23 - 1.23]
GSE16131-GPL96	Blood cancer	Follicular lymphoma	Overall Survival	HG-U133A	214214_s_at	180	0.343015	0.80 [0.51 - 1.27]
GSE16131-GPL96	Blood cancer	Follicular lymphoma	Overall Survival	HG-U133A	208910_s_at	180	0.236042	0.78 [0.52 - 1.18]
GSE2658	Blood cancer	Multiple myeloma	Disease Specific Survival	HG-U133_Plus_2	214214_s_at	559	0.294184	1.28 [0.81 - 2.02]
GSE2658	Blood cancer	Multiple myeloma	Disease Specific Survival	HG-U133_Plus_2	208910_s_at	559	0.158587	1.33 [0.89 - 1.97]
GSE4271-GPL96	Brain cancer	Astrocytoma	Overall Survival	HG-U133A	208910_s_at	77	0.0793001	1.88 [0.93 - 3.80]
GSE4271-GPL96	Brain cancer	Astrocytoma	Overall Survival	HG-U133A	214214_s_at	77	0.0920103	1.87 [0.90 - 3.85]
GSE7696	Brain cancer	Glioblastoma	Overall Survival	HG-U133_Plus_2	214214_s_at	70	0.190584	0.67 [0.37 - 1.22]

GSE7696	Brain cancer	Glioblastoma	Overall Survival	HG-U133_Plus_2	208910_s_at	70	0.381773	0.82 [0.52 - 1.29]
GSE4412-GPL96	Brain cancer	Glioma	Overall Survival	HG-U133A	208910_s_at	74	0.0331974	0.35 [0.13 - 0.92]
GSE4412-GPL96	Brain cancer	Glioma	Overall Survival	HG-U133A	214214_s_at	74	0.626016	0.76 [0.25 - 2.28]
GSE16581	Brain cancer	Meningioma	Overall Survival	HG-U133_Plus_2	214214_s_at	67	0.407419	2.40 [0.30 - 19.16]
GSE16581	Brain cancer	Meningioma	Overall Survival	HG-U133_Plus_2	208910_s_at	67	0.432774	2.42 [0.27 - 22.09]
GSE22138	Eye cancer	Uveal melanoma	Distant Metastasis Free Survival	HG-U133_Plus_2	214214_s_at	63	0.185756	1.50 [0.82 - 2.71]
GSE22138	Eye cancer	Uveal melanoma	Distant Metastasis Free Survival	HG-U133_Plus_2	208910_s_at	63	0.184453	1.41 [0.85 - 2.33]
GSE2837	Head and neck cancer	Squamous cell carcinoma	Relapse Free Survival	U133_X3P	Hs.78614.3.A1_3p_a_at	28	0.963406	0.99 [0.62 - 1.57]
GSE9891	Ovarian cancer		Overall Survival	HG-U133_Plus_2	208910_s_at	278	0.00451267	0.59 [0.41 - 0.85]
GSE9891	Ovarian cancer		Overall Survival	HG-U133_Plus_2	214214_s_at	278	0.0131126	0.64 [0.45 - 0.91]
DUKE-OC	Ovarian cancer		Overall Survival	HG-U133A	214214_s_at	133	0.466614	0.89 [0.64 - 1.22]
DUKE-OC	Ovarian cancer		Overall Survival	HG-U133A	208910_s_at	133	0.515996	0.94 [0.77 - 1.14]
GSE8841	Ovarian cancer		Overall Survival	G4100A	18486	81	0.708878	0.85 [0.37 - 1.97]
GSE26712	Ovarian cancer		Overall Survival	HG-U133_Plus_2	214214_s_at	185	0.809756	1.02 [0.87 - 1.19]
GSE26712	Ovarian cancer		Overall Survival	HG-U133_Plus_2	208910_s_at	185	0.897145	0.99 [0.84 - 1.17]
GSE26712	Ovarian cancer		Disease Free Survival	HG-U133_Plus_2	208910_s_at	185	0.881351	0.99 [0.85 - 1.15]
GSE26712	Ovarian cancer		Disease Free Survival	HG-U133_Plus_2	214214_s_at	185	0.949237	1.00 [0.87 - 1.16]
GSE17260	Ovarian cancer		Progression Free Survival	G4112A	A_23_P370434	110	0.664848	1.08 [0.76 - 1.55]
GSE17260	Ovarian cancer		Overall Survival	G4112A	A_23_P370434	110	0.456883	1.19 [0.76 - 1.87]
GSE14764	Ovarian cancer		Overall Survival	HG-U133A	214214_s_at	80	0.504511	1.22 [0.68 - 2.20]
GSE14764	Ovarian cancer		Overall Survival	HG-U133A	208910_s_at	80	0.787616	1.09 [0.60 - 1.98]
E-DKFZ-1	Renal cell carcinoma		Overall Survival	A-RZPD-20	28429	59	0.994155	0.99 [0.14 - 6.86]
GSE19234	Skin cancer	Melanoma	Overall Survival	HG-U133_Plus_2	208910_s_at	38	0.00638691	5.58 [1.62 - 19.17]
GSE19234	Skin cancer	Melanoma	Overall Survival	HG-U133_Plus_2	214214_s_at	38	0.0200671	4.04 [1.25 - 13.11]

GSE30929	Soft tissue cancer	Liposarcoma	Distant Recurrence Free Survival	HG-U133A	208910_s_at	140	0.324408	1.35 [0.75 - 2.43]
GSE30929	Soft tissue cancer	Liposarcoma	Distant Recurrence Free Survival	HG-U133A	214214_s_at	140	0.351669	1.29 [0.76 - 2.19]

Supplementary Table S9. Gene ontology (GO) terms obtained from genes positively correlated with *C1QBP*.

Gene Ontology term	Cluster frequency	Genome frequency	Corrected *p*-value
**Biological Process**			
ribosome biogenesis	19 of 67 genes, 28.4%	291 of 19782 genes, 1.5%	9.32E-17
ribonucleoprotein complex biogenesis	20 of 67 genes, 29.9%	456 of 19782 genes, 2.3%	2.17E-14
translation	21 of 67 genes, 31.3%	645 of 19782 genes, 3.3%	1.19E-12
peptide biosynthetic process	21 of 67 genes, 31.3%	670 of 19782 genes, 3.4%	2.53E-12
cellular nitrogen compound metabolic process	53 of 67 genes, 79.1%	6598 of 19782 genes, 33.4%	1.57E-11
amide biosynthetic process	21 of 67 genes, 31.3%	805 of 19782 genes, 4.1%	9.25E-11
peptide metabolic process	21 of 67 genes, 31.3%	819 of 19782 genes, 4.1%	1.29E-10
RNA processing	21 of 67 genes, 31.3%	927 of 19782 genes, 4.7%	1.39E-09
ribosomal large subunit biogenesis	9 of 67 genes, 13.4%	73 of 19782 genes, 0.4%	2.35E-09
ncRNA metabolic process	17 of 67 genes, 25.4%	566 of 19782 genes, 2.9%	3.49E-09
gene expression	46 of 67 genes, 68.7%	5502 of 19782 genes, 27.8%	3.54E-09
mitochondrial gene expression	11 of 67 genes, 16.4%	160 of 19782 genes, 0.8%	5.11E-09
cellular amide metabolic process	21 of 67 genes, 31.3%	1076 of 19782 genes, 5.4%	2.33E-08
ncRNA processing	14 of 67 genes, 20.9%	385 of 19782 genes, 1.9%	2.91E-08
rRNA processing	11 of 67 genes, 16.4%	207 of 19782 genes, 1.0%	8.33E-08
cellular component organization or biogenesis	48 of 67 genes, 71.6%	6577 of 19782 genes, 33.2%	0.000000121
nucleobase-containing compound metabolic process	45 of 67 genes, 67.2%	5904 of 19782 genes, 29.8%	0.000000259
cellular component biogenesis	33 of 67 genes, 49.3%	3226 of 19782 genes, 16.3%	0.000000332
cellular metabolic process	60 of 67 genes, 89.6%	10759 of 19782 genes, 54.4%	0.000000451
rRNA metabolic process	11 of 67 genes, 16.4%	243 of 19782 genes, 1.2%	0.000000461
mitochondrial translation	9 of 67 genes, 13.4%	135 of 19782 genes, 0.7%	0.000000643
organonitrogen compound biosynthetic process	25 of 67 genes, 37.3%	1882 of 19782 genes, 9.5%	0.000000675
heterocycle metabolic process	45 of 67 genes, 67.2%	6066 of 19782 genes, 30.7%	0.000000687
cellular aromatic compound metabolic process	45 of 67 genes, 67.2%	6112 of 19782 genes, 30.9%	0.0000009
ribosome assembly	7 of 67 genes, 10.4%	67 of 19782 genes, 0.3%	0.00000235
organic cyclic compound metabolic process	45 of 67 genes, 67.2%	6323 of 19782 genes, 32.0%	0.000003
macromolecule metabolic process	55 of 67 genes, 82.1%	9507 of 19782 genes, 48.1%	0.00000734
nucleic acid metabolic process	40 of 67 genes, 59.7%	5225 of 19782 genes, 26.4%	0.00000813
nitrogen compound metabolic process	57 of 67 genes, 85.1%	10285 of 19782 genes, 52.0%	0.0000103
translational elongation	8 of 67 genes, 11.9%	135 of 19782 genes, 0.7%	0.0000152
primary metabolic process	58 of 67 genes, 86.6%	10780 of 19782 genes, 54.5%	0.0000182
metabolic process	60 of 67 genes, 89.6%	11606 of 19782 genes, 58.7%	0.0000222
posttranscriptional regulation of gene expression	13 of 67 genes, 19.4%	570 of 19782 genes, 2.9%	0.000043
translational termination	7 of 67 genes, 10.4%	104 of 19782 genes, 0.5%	0.0000521

organic substance metabolic process	58 of 67 genes, 86.6%	11123 of 19782 genes, 56.2%	0.0000805
RNA localization	9 of 67 genes, 13.4%	235 of 19782 genes, 1.2%	0.0000814
mRNA metabolic process	15 of 67 genes, 22.4%	840 of 19782 genes, 4.2%	0.0000874
ribonucleoprotein complex assembly	9 of 67 genes, 13.4%	241 of 19782 genes, 1.2%	0.0001
cellular nitrogen compound biosynthetic process	37 of 67 genes, 55.2%	4985 of 19782 genes, 25.2%	0.00012
ribonucleoprotein complex subunit organization	9 of 67 genes, 13.4%	255 of 19782 genes, 1.3%	0.00016
regulation of translation	10 of 67 genes, 14.9%	340 of 19782 genes, 1.7%	0.00017
establishment of protein localization to organelle	12 of 67 genes, 17.9%	534 of 19782 genes, 2.7%	0.00017
mitochondrial translational elongation	6 of 67 genes, 9.0%	88 of 19782 genes, 0.4%	0.00044
regulation of cellular amide metabolic process	10 of 67 genes, 14.9%	392 of 19782 genes, 2.0%	0.00064
macromolecule biosynthetic process	36 of 67 genes, 53.7%	5059 of 19782 genes, 25.6%	0.00068
cellular component organization	41 of 67 genes, 61.2%	6402 of 19782 genes, 32.4%	0.00098
cellular macromolecule biosynthetic process	35 of 67 genes, 52.2%	4920 of 19782 genes, 24.9%	0.00115
regulation of mitochondrial translation	4 of 67 genes, 6.0%	25 of 19782 genes, 0.1%	0.00123
protein-containing complex subunit organization	22 of 67 genes, 32.8%	2197 of 19782 genes, 11.1%	0.00146
nucleocytoplasmic transport	9 of 67 genes, 13.4%	342 of 19782 genes, 1.7%	0.00188
nuclear transport	9 of 67 genes, 13.4%	345 of 19782 genes, 1.7%	0.00202
positive regulation of translation	6 of 67 genes, 9.0%	120 of 19782 genes, 0.6%	0.00278
cellular biosynthetic process	39 of 67 genes, 58.2%	6112 of 19782 genes, 30.9%	0.00285
telomere maintenance via telomerase	5 of 67 genes, 7.5%	70 of 19782 genes, 0.4%	0.00335
ribosomal large subunit assembly	4 of 67 genes, 6.0%	32 of 19782 genes, 0.2%	0.00345
protein localization to nuclear body	3 of 67 genes, 4.5%	10 of 19782 genes, 0.1%	0.00375
positive regulation of establishment of protein localization to telomere	3 of 67 genes, 4.5%	10 of 19782 genes, 0.1%	0.00375
protein localization to Cajal body	3 of 67 genes, 4.5%	10 of 19782 genes, 0.1%	0.00375
RNA metabolic process	33 of 67 genes, 49.3%	4693 of 19782 genes, 23.7%	0.00406
organic substance biosynthetic process	39 of 67 genes, 58.2%	6206 of 19782 genes, 31.4%	0.00431
regulation of establishment of protein localization to telomere	3 of 67 genes, 4.5%	11 of 19782 genes, 0.1%	0.00515
biosynthetic process	39 of 67 genes, 58.2%	6280 of 19782 genes, 31.7%	0.00593
regulation of establishment of protein localization to chromosome	3 of 67 genes, 4.5%	12 of 19782 genes, 0.1%	0.00685
positive regulation of protein localization to chromosome, telomeric region	3 of 67 genes, 4.5%	12 of 19782 genes, 0.1%	0.00685
protein localization to nucleoplasm	3 of 67 genes, 4.5%	12 of 19782 genes, 0.1%	0.00685
telomere maintenance via telomere lengthening	5 of 67 genes, 7.5%	81 of 19782 genes, 0.4%	0.00689
positive regulation of cellular amide metabolic process	6 of 67 genes, 9.0%	141 of 19782 genes, 0.7%	0.00705
protein localization to organelle	13 of 67 genes, 19.4%	899 of 19782 genes, 4.5%	0.0072

cellular protein complex disassembly	7 of 67 genes, 10.4%	217 of 19782 genes, 1.1%	0.0074
RNA-dependent DNA biosynthetic process	5 of 67 genes, 7.5%	83 of 19782 genes, 0.4%	0.00777
**Molecular Function**			
RNA binding	39 of 67 genes, 58.2%	1654 of 19782 genes, 8.4%	4.88E-23
nucleic acid binding	40 of 67 genes, 59.7%	4128 of 19782 genes, 20.9%	6.73E-10
heterocyclic compound binding	46 of 67 genes, 68.7%	6068 of 19782 genes, 30.7%	2.33E-08
organic cyclic compound binding	46 of 67 genes, 68.7%	6155 of 19782 genes, 31.1%	3.95E-08
structural constituent of ribosome	8 of 67 genes, 11.9%	173 of 19782 genes, 0.9%	0.0000169
ribonucleoprotein complex binding	6 of 67 genes, 9.0%	132 of 19782 genes, 0.7%	0.00078
mRNA binding	7 of 67 genes, 10.4%	243 of 19782 genes, 1.2%	0.00249
binding	64 of 67 genes, 95.5%	15184 of 19782 genes, 76.8%	0.00424
translation elongation factor activity	3 of 67 genes, 4.5%	21 of 19782 genes, 0.1%	0.00657
**Cellular Component**			
organelle part	61 of 67 genes, 91.0%	9599 of 19782 genes, 48.5%	2.33E-11
mitochondrial part	23 of 67 genes, 34.3%	1046 of 19782 genes, 5.3%	3.45E-11
intracellular organelle part	60 of 67 genes, 89.6%	9298 of 19782 genes, 47.0%	3.95E-11
mitochondrion	26 of 67 genes, 38.8%	1593 of 19782 genes, 8.1%	5.52E-10
ribonucleoprotein complex	20 of 67 genes, 29.9%	858 of 19782 genes, 4.3%	6.68E-10
protein-containing complex	45 of 67 genes, 67.2%	5397 of 19782 genes, 27.3%	1.99E-09
membrane-enclosed lumen	44 of 67 genes, 65.7%	5235 of 19782 genes, 26.5%	3.54E-09
organelle lumen	44 of 67 genes, 65.7%	5235 of 19782 genes, 26.5%	3.54E-09
intracellular organelle lumen	44 of 67 genes, 65.7%	5235 of 19782 genes, 26.5%	3.54E-09
intracellular membrane-bounded organelle	62 of 67 genes, 92.5%	11017 of 19782 genes, 55.7%	5.3E-09
membrane-bounded organelle	65 of 67 genes, 97.0%	12527 of 19782 genes, 63.3%	6.68E-09
mitochondrial matrix	15 of 67 genes, 22.4%	469 of 19782 genes, 2.4%	6.69E-09
intracellular non-membrane-bounded organelle	39 of 67 genes, 58.2%	4229 of 19782 genes, 21.4%	1.03E-08
non-membrane-bounded organelle	39 of 67 genes, 58.2%	4239 of 19782 genes, 21.4%	1.11E-08
organelle envelope	21 of 67 genes, 31.3%	1192 of 19782 genes, 6.0%	3.23E-08
envelope	21 of 67 genes, 31.3%	1192 of 19782 genes, 6.0%	3.23E-08
cytoplasm	62 of 67 genes, 92.5%	11606 of 19782 genes, 58.7%	9.67E-08
mitochondrial inner membrane	14 of 67 genes, 20.9%	476 of 19782 genes, 2.4%	9.79E-08
nucleolus	18 of 67 genes, 26.9%	932 of 19782 genes, 4.7%	0.000000208
mitochondrial membrane	16 of 67 genes, 23.9%	706 of 19782 genes, 3.6%	0.000000214
nuclear part	38 of 67 genes, 56.7%	4483 of 19782 genes, 22.7%	0.000000305
organelle inner membrane	14 of 67 genes, 20.9%	535 of 19782 genes, 2.7%	0.000000442
mitochondrial envelope	16 of 67 genes, 23.9%	748 of 19782 genes, 3.8%	0.000000491
organelle	65 of 67 genes, 97.0%	13632 of 19782 genes, 68.9%	0.0000012
ribosome	10 of 67 genes, 14.9%	242 of 19782 genes, 1.2%	0.00000149
intracellular organelle	63 of 67 genes, 94.0%	12805 of 19782 genes, 64.7%	0.00000286
ribosomal subunit	9 of 67 genes, 13.4%	196 of 19782 genes, 1.0%	0.00000354
intracellular	66 of 67 genes, 98.5%	14601 of 19782 genes, 73.8%	0.0000062

intracellular part	66 of 67 genes, 98.5%	14601 of 19782 genes, 73.8%	0.0000062
cytoplasmic part	54 of 67 genes, 80.6%	9723 of 19782 genes, 49.2%	0.0000185
nucleus	46 of 67 genes, 68.7%	7399 of 19782 genes, 37.4%	0.0000359
nuclear lumen	32 of 67 genes, 47.8%	4093 of 19782 genes, 20.7%	0.00011
cytosolic part	8 of 67 genes, 11.9%	255 of 19782 genes, 1.3%	0.00041
cytosolic large ribosomal subunit	5 of 67 genes, 7.5%	65 of 19782 genes, 0.3%	0.00048
large ribosomal subunit	6 of 67 genes, 9.0%	123 of 19782 genes, 0.6%	0.00066
cytosol	33 of 67 genes, 49.3%	5088 of 19782 genes, 25.7%	0.0052
mitochondrial protein complex	7 of 67 genes, 10.4%	272 of 19782 genes, 1.4%	0.00658
cytosolic ribosome	5 of 67 genes, 7.5%	117 of 19782 genes, 0.6%	0.00852

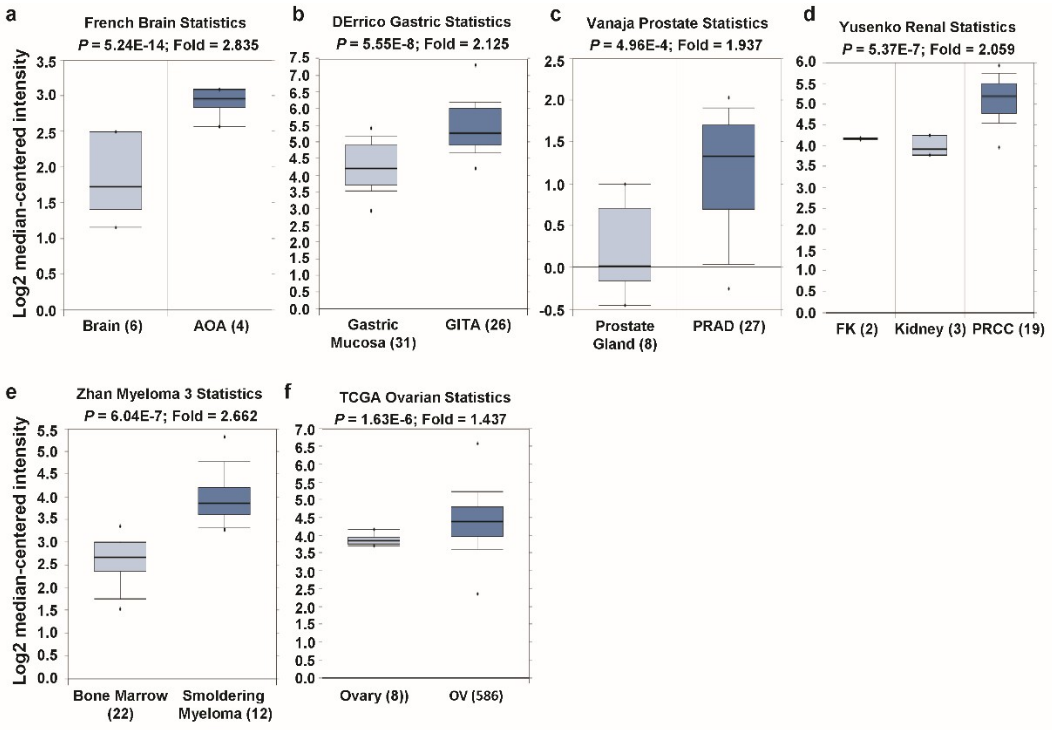

Supplementary Figure S1. *C1QBP* expression analysis in different cancer types (Oncomine database). The box plot comparing specific *C1QBP* expression in normal (left plot) and cancer tissue (right plot) was derived from Oncomine database. The fold change of *C1QBP* in various cancer types was identified from our analyses. The analysis was shown in AOA relative to normal brain **(a)**, in GITA relative to Gastric Mucosa **(a)**, in PRAD relative to normal Prostate Gland (**c**), in PRCC relative to normal Kidney (**d**), in Smoldering Myeloma relative to normal Bone Marrow (**e**), in OV relative to normal Ovary (**f**). (Abbreviations: AOA-Anaplastic Oligoastrocytoma; FK-Fetal Kidney; SBC-Superficial Bladder Cancer; GITA-Gastric Intestinal Type Adenocarcinoma; PRAD-PAAD-Pancreatic adenocarcinoma; PRCC-Papillary Renal Cell Carcinoma; NPCBL- Naive Pregerminal Center B-Lymphocyte; OV-Ovarian Serous Cystadenocarcinoma).

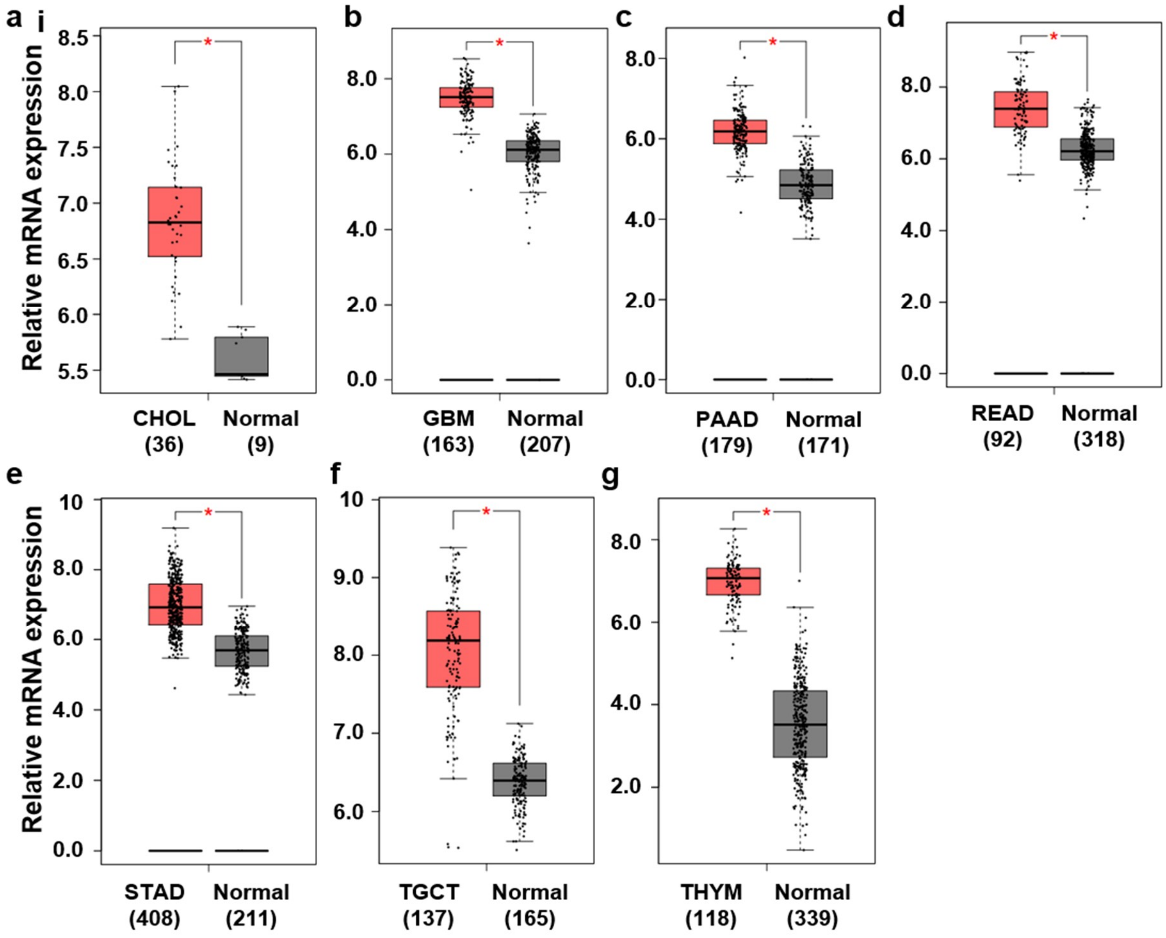

Supplementary Figure S2. *C1QBP* expression analysis in different cancer types (TCGA database). Expression of *C1QBP* gene in the Cancer Genome Atlas (TCGA) database. Box plots showing the *C1QBP* mRNA expression in various tumor (T) and their normal (N) tissues, using data from the TCGA database through GEPIA web (**a-g**). The threshold was designed with specific parameters; *: *p*-value = 0.01, fold change = 2. (Abbreviations: CHOL-Cholangio carcinoma; DLBC-Lymphoid Neoplasm Diffuse Large B-cell Lymphoma; GBM-Glioblastoma multiforme; PAAD-Pancreatic adenocarcinoma; READ-Rectum adenocarcinoma; STAD-Stomach adenocarcinoma; TGCT-Testicular Germ Cell Tumors; THYM-Thymoma).

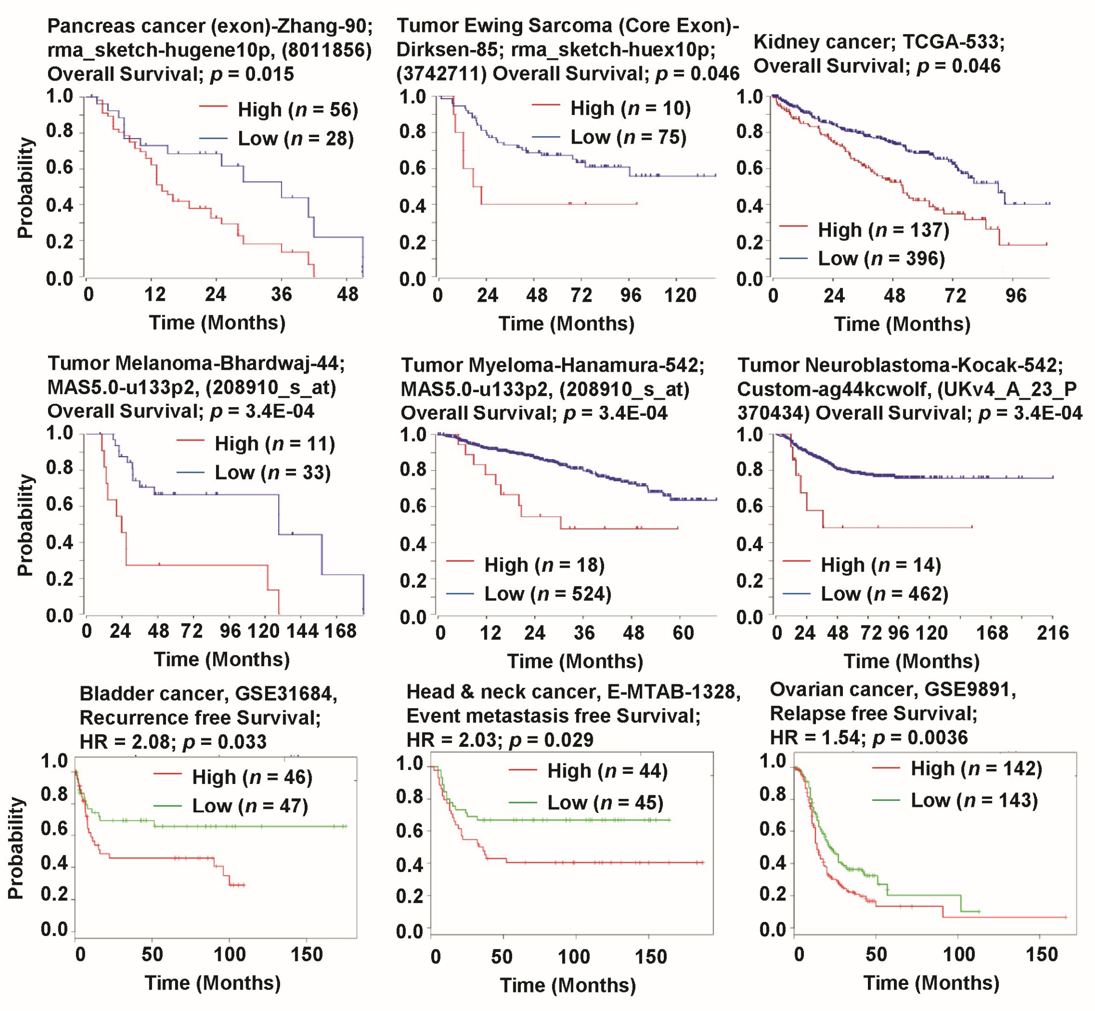

Supplementary Figure S3. Correlation of *C1QBP* gene expression with various cancer prognosis (R2: Kaplan Meier Scanner and SurvExpress). The survival curve comparing the patient with high (red) and low (blue/green) expression of *C1QBP* was plotted from R2: Kaplan Meier Scanner and SurvExpress database in pancreas, lymphoma, sarcoma, kidney, melanoma, myeloma, brain, bladder, head & neck, and ovarian cancers. Survival curve analysis was conducted using a threshold Cox *p*-value < 0.05.

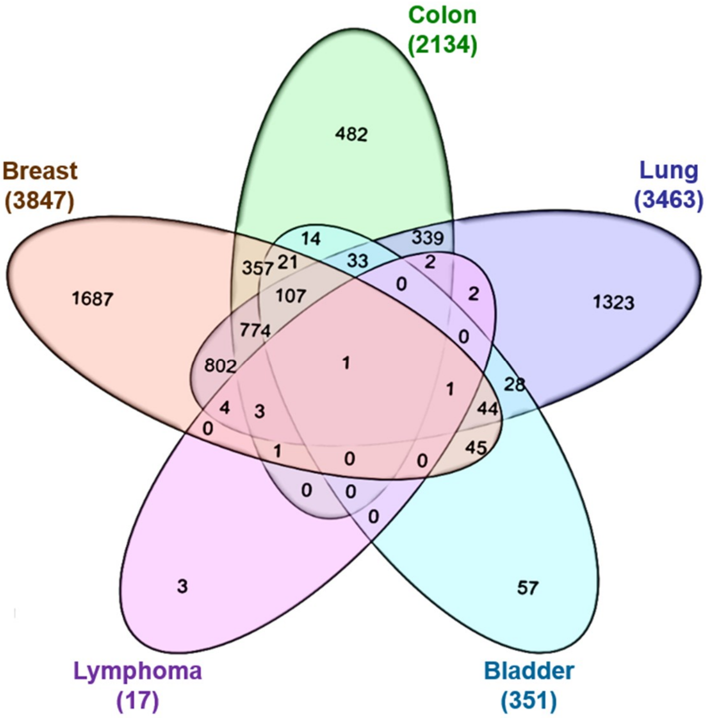

Supplementary Figure S4. Analysis of negatively correlated genes of *C1QBP*. Venn diagram of genes negatively correlated to *C1QBP*, showing coincident genes in Breast, Colon, Lung, Bladder, and Lymphoma cancers.
